# Systematic comparison and assessment of RNA-seq procedures for gene expression quantitative analysis

**DOI:** 10.1038/s41598-020-76881-x

**Published:** 2020-11-12

**Authors:** Luis A. Corchete, Elizabeta A. Rojas, Diego Alonso-López, Javier De Las Rivas, Norma C. Gutiérrez, Francisco J. Burguillo

**Affiliations:** 1 Hematology Department, University Hospital, 37007 Salamanca, Spain; 2 Cancer Research Center (CiC-IBMCC, CSIC/USAL), Consejo Superior de Investigaciones Científicas (CSIC) and University of Salamanca (USAL), 37007 Salamanca, Spain; 3 Institute of Biomedical Research of Salamanca (IBSAL), 37007 Salamanca, Spain; 4 Faculty of Pharmacy, University of Salamanca, 37007 Salamanca, Spain; 5 Center for Biomedical Research in Network of Cancer (CIBERONC), Salamanca, Spain

**Keywords:** Cancer genetics, Gene expression, Computational biology and bioinformatics

## Abstract

RNA-seq is currently considered the most powerful, robust and adaptable technique for measuring gene expression and transcription activation at genome-wide level. As the analysis of RNA-seq data is complex, it has prompted a large amount of research on algorithms and methods. This has resulted in a substantial increase in the number of options available at each step of the analysis. Consequently, there is no clear consensus about the most appropriate algorithms and pipelines that should be used to analyse RNA-seq data. In the present study, 192 pipelines using alternative methods were applied to 18 samples from two human cell lines and the performance of the results was evaluated. Raw gene expression signal was quantified by non-parametric statistics to measure precision and accuracy. Differential gene expression performance was estimated by testing 17 differential expression methods. The procedures were validated by qRT-PCR in the same samples. This study weighs up the advantages and disadvantages of the tested algorithms and pipelines providing a comprehensive guide to the different methods and procedures applied to the analysis of RNA-seq data, both for the quantification of the raw expression signal and for the differential gene expression.

## Introduction

In recent years, RNA-seq has emerged as an alternative method to that of classic microarrays for transcriptome analysis^1–4^. Compared with microarrays, RNA-seq enables the study of novel transcripts and offers higher resolution, a better range of detection and lower technical variability^5,6^. RNA-seq also offers a high degree of agreement with other techniques considered as the gold standard in transcriptomics such as qRT-PCR, both at absolute and relative gene expression measurement levels^7^. All these facts have led to a great expansion of RNA-seq, becoming the first choice in transcriptomic analysis for many scientists. Nevertheless, its widespread use has generated a large amount of research on algorithms and methods that has eventually produced a lack of consensus about how to analyse RNA-seq data. Thereby, in the last decade, many different algorithms and pipelines have been proposed, but there is much debate about which approaches provide the most precise and accurate results. Thus, further research to compare these methods remains necessary.

RNA-seq data analysis typically involves several steps: trimming, alignment, counting and normalization of the sequenced reads, and, very often, differential expression (DE) analysis across conditions. Trimming is used to increase reads mapping rate through the elimination of the adapter sequences and the removal of poor-quality nucleotides. It must be employed non-aggressively, together with a wisely chosen read length, to avoid unpredictable changes in gene expression^8^ and transcriptome assembly^9^. The beneficial effects of this process have been evaluated in reference-based^10,11^ and de novo^12,13^ RNA-seq analyses. The alignment to a reference genome or transcriptome is normally the second step in the RNA-seq workflow and has been widely evaluated by many authors^14–19^. Once the reads have been mapped, they must be assigned to a gene or a transcript, in a process known as counting or quantification. This is followed by a normalization procedure to remove possible sequencing bias. Since counting followed by normalization is a crucial component of RNA-seq data analysis, several methods have been developed and many comparative studies evaluating their suitability have been published^20–28^. The final step in most RNA-seq studies is DE analysis. The main concern at this point is how to accurately detect differentially expressed genes (DEGs) between two or more conditions. For that purpose, a large number of tools have been developed to facilitate the analysis, and thereby to construct lists of significant DEGs^29–38^. Due to the impact of this final step, the algorithms involved in DEG detection have been compared in many publications where authors analyse the pros and cons of applying different algorithms^23,29,39–48^.

In this scenario, the major challenge in RNA-seq analysis is that the different steps must be sequentially combined in a complete workflow or pipeline and users have to choose between many possible methodological approaches and options. This shows the complexity of RNA-seq analysis and it is one of the most critical points to obtain accurate results both at raw and DE expression levels. Many possible combinations of the current algorithms in RNA-seq have been comparatively analysed to help decide the best workflow, but their performance remains under discussion. For example, Nookaew et al.^49^ investigated several workflows to pass from RNA-seq reads to differential gene expression (DGE) in *Saccharomyces cerevisiae*. Seyednasrollah et al.^43^ compared software packages for DE using two public data sets. Teng et al.^50^ suggested new metrics to assess differences between competing pipelines and Williams et al.^51^ evaluated many workflows for DE in human monocytes sequenced as 51 bp single-end reads.

**Figure 1:**
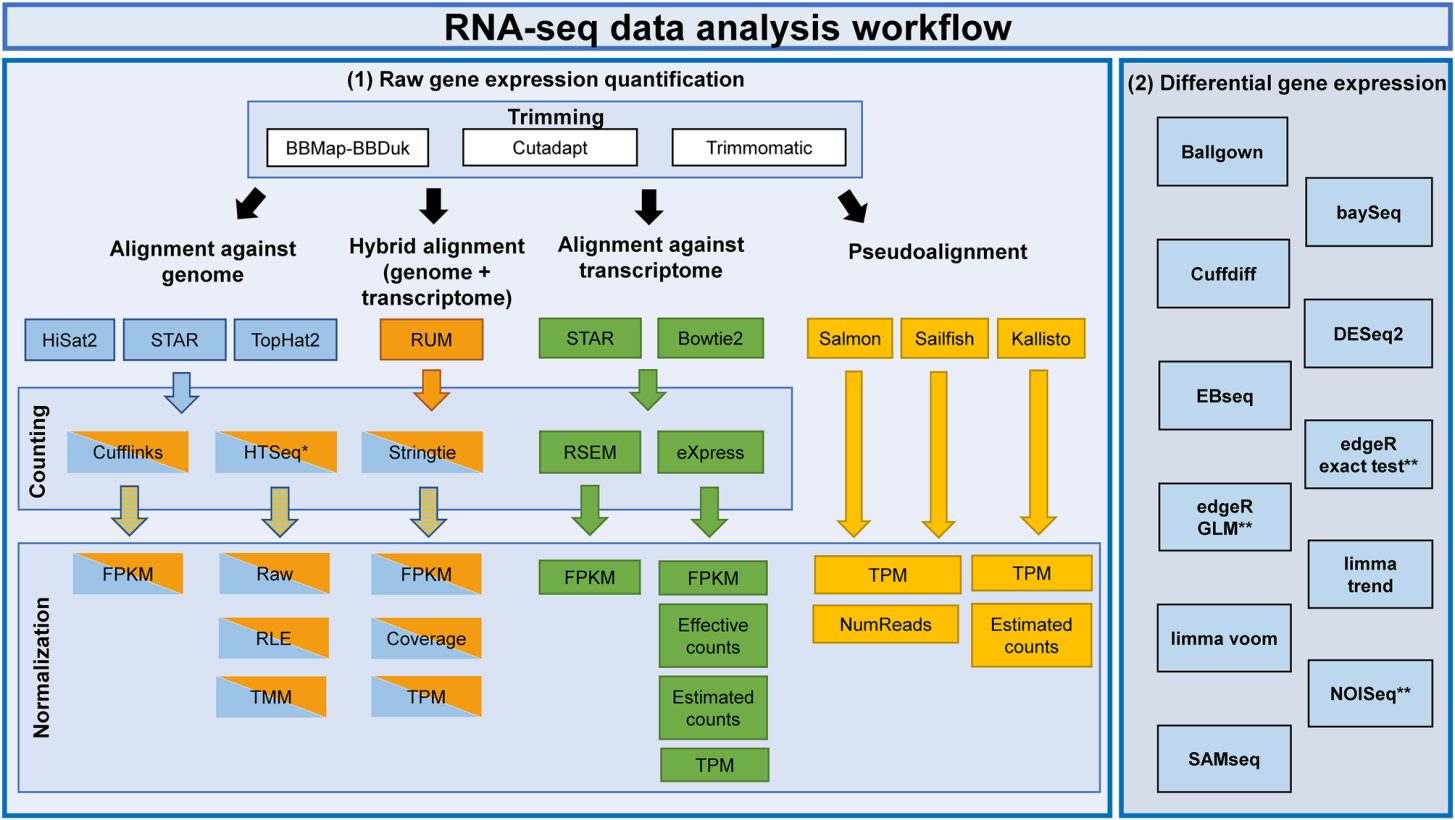
RNA-seq analysis workflow. Left panel (1) represents the raw gene expression quantification workflow. Every box contains the algorithms and methods used for the RNA-seq analysis at trimming, alignment, counting, normalization and pseudoalignment levels. The right panel (2) represents the algorithms used for the differential gene expression quantification. *HTSeq was performed in two modes: union and intersection-strict. **EdgeR exact test, *edgeR GLM* and *NOISeq* have internally three normalization techniques that were evaluated separately.

**Figure 2:**
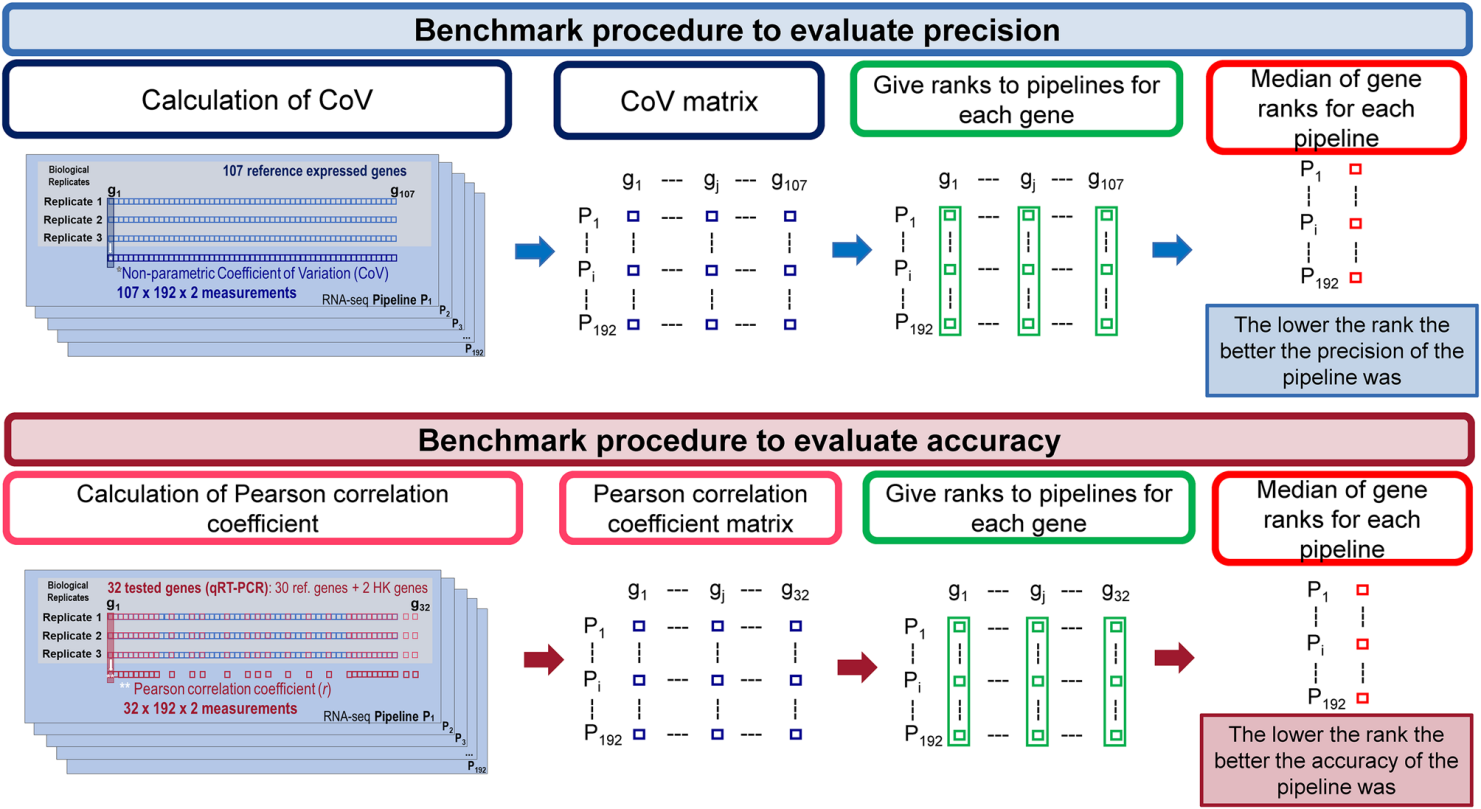
Benchmark procedure to evaluate precision and accuracy. Description of the procedure to evaluate the precision (top) and the accuracy (bottom) in the RNA-seq analysis.

Facing with the problems described, the present work aims to evaluate 192 alternative methodological pipelines using Illumina HiSeq 2500 paired-end RNA-seq data measuring its accuracy and precision at raw gene expression quantification level (RGEQ). These pipelines were constructed performing all the possible combinations of 3 trimming algorithms, 5 aligners, 6 counting methods, 3 pseudoaligners and 8 normalization approaches. We also evaluated the performance of 17 DE methods using the results of the top 10 ranked RGEQ pipelines. All these algorithms and methods were selected based on their popularity as they are used in dozens of scientific publications and can be easily implemented into a pipeline (Fig. 1). As samples, we used data from two multiple myeloma (MM) cell lines, treated with two different drugs as putative therapeutic molecules. A benchmark evaluation protocol for the accuracy and precision of each pipeline was set up, based in the experimental testing of 32 genes by qRT-PCR and the detection of 107 house-keeping reference expressed genes (presented in Fig. 2 and later described in more detail).

## Methods

### RNA-seq data

We used two extensively characterized MM cell lines, KMS12-BM (cell line A [CLA]) and JJN-3 (cell line B [CLB]), as previously described^52^. Both cell lines were treated with Amiloride at 0.1 mM (KMS12-BM) and 0.4 mM (JJN-3) for 24 h (treatment 1 [T1]), with TG003 at 50 µM (both KMS12-BM and JJN-3) for 24 h (treatment 2 [T2]), and dimethyl-sulfoxide (DMSO) (treatment 0 [T0]) was used as negative control. All the experiments were done by triplicate, making up a total of 18 samples. RNA was extracted using the RNeasy Plus Mini kit (QIAGEN, Hilden, Germany). The RNA integrity was assessed with an Agilent 2100 Bioanalyzer (Agilent Technologies). The corresponding 18 RNA libraries were constructed following the *TruSeq Strand-Specific RNA sequencing library* protocol from Illumina (https://support.illumina.com/downloads/truseq_stranded_total_rna_sample_preparation_guide_15031048.html). The RNA libraries were sequenced in a HiSeq 2500 system at Lifesequencing S.L. (Valencia, Spain). Paired-end reads of 101 base pairs (within a range of 36,240,231–77,906,369 total reads), were generated using this platform (Supplementary Table S1). The quality of the resulting sequences in FASTQ format was assessed using the *FASTQC* (v0.11.3) tool^53^.

### Gene expression validation using qRT-PCR

To determine qRT-PCR candidate genes we first selected in our dataset 1181 genes expressed in 32 healthy tissues from the RNA-seq data published by Uhlen et al.^54^. We then performed a filtering process removing all genes with < 4 gene expression units (alignments, FPKM, TPM, etc. depending on the normalization method) in the six DMSO treated samples for each pipeline. This process resulted in the choice of 107 genes that satisfied this filtering criterion in all the 192 pipelines (Supplementary Table S2). As these 107 were constitutively expressed, both in 32 healthy tissues and in our two cell lines, we considered them as our gene expression reference set or housekeeping gene set (HKg). We selected from this first list 30 genes, 10 with the highest and 10 with the lowest median non-parametric coefficient of variation (CoV)^55^, and 10 random genes from the mid-area. Additionally, we selected two commonly used housekeeping genes in RNA studies: *GAPDH* and *ACTB*, generating a second list of 32 genes. Starting from the same samples used in RNA-seq, 1 µg of total RNA was reverse transcribed to cDNA using oligo dT from SuperScript First-Strand Synthesis System for RT-PCR (Thermo Fisher Scientific). Taqman qRT-PCR mRNA assays (Applied Biosystems) were carried out in duplicate on these 32 genes that are highlighted in the Supplementary Table S2. Oligonucleotide probes used for the qRT-PCR assays are reported in the Supplementary Table S3.

To measure DGE by qRT-PCR we used the ΔCt method, calculated as:

$$ \Delta Ct = Ct_{Control gene} { }{-}{ }Ct_{Target gene} $$

Three normalization approaches were tested: a) *Endogenous control normalization*, where endogenous control was calculated as the mean of *GAPDH* and *ACTB* Ct values for each sample, b) *Global median normalization*, in which the normalization factor was calculated using the median value for genes with Ct < 35 for each sample, and c) *Most stable gene*, that was determined using the 4 algorithms available in the RefFinder webtool, *BestKeeper*^56^, *NormFinder*^57^, *Genorm*^58^ and *the comparative delta-Ct method*^59^. The gene *ECHS1* was ranked the top position and it was considered as the most stable in our dataset. We detected bias on *GAPDH* and *ACTB* genes expression due to the drug treatments. This bias was an under-expression of these genes in the treatment conditions as it is shown in Supplementary Fig. S1. In view of this fact, we rejected the (a) normalization method. Regarding the (b) and (c) methods, both performed equally well but the (c) method looked more robust than (b) as it captures better the Ct values dispersion inside each sample. Finally, we chose the (b) method (*Global median normalization*) for our downstream analysis.

### RNA-seq analysis

#### Trimming

We decided to apply the trimming procedure to eliminate adapter sequences present in our data and to improve read quality from the FASTQ files. Adapter removal and quality trimming were carried out using *Trimmomatic*^60^ (v.0.35), *Cutadapt*^61^ (v.1.12) and *BBDuk* (v.Oct., 23, 2015), the last included in the BBTools suite (https://sourceforge.net/projects/bbmap/). In all cases only reads with a Phred quality score > 20 and read length > 50 bp were selected for downstream analysis. The statistical calculations for the comparisons among the trimming algorithms regarding the reads mapping rate and the surviving reads were performed using the Kruskal–Wallis test, followed by the Dunn’s post-hoc test in the *dunn.test*^62^ (v.1.3.5) package in R^63^ (v.3.5.1). Details about each trimming algorithm are given in Supplementary Note S1.

#### Alignment

We next evaluated the genome-based alignment methods *Tophat2*^16^ (v.2.1.0), *STAR*^18^ (v.2.5.3a) and *Hisat2*^64^ (v.2.0.0). The Human genome version GRCh37 (hg19) from Ensembl^65^ was used as the reference genome. Transcriptome alignment methods represented by *Bowtie2*^66^ (v.2.2.6) and *STAR* were also tested against the Ensembl (v82) transcriptome. Finally, hybrid methods, which combine both types of alignment, were represented by *RUM*^19^ (v.2.0.5_06) that used its own hg19 reference. A pre-processing step of adding Ns letters to the incomplete reads was needed in *RUM* since paired reads with different lengths were not allowed. BAM files from all the methods were sorted by read name and genome position using *samtools*^67^ (v.1.3.1) and unmapped reads were discarded. The statistical calculations concerning the unmapped reads for each algorithm were made using the Kruskal–Wallis test, followed by the Dunn’s post-hoc test in the *dunn.test* package. Details about each aligner are given in Supplementary Note S1.

#### Counting and normalization

The number of alignments mapped to each gene was counted using as reference a gene transfer format (GTF) file from Ensembl (v.82). We used six counting methods: *Cufflinks*^68^ (v.2.2.1), *eXpress*^69^ (v.1.5.1), *HTSeq*^70^ (v.0.6.1p1) using both the *Intersection-Strict* and the *Union* approaches, *RSEM*^27^ (v.1.2.31) and *Stringtie*^71^ (v.1.3.3b). Gene expression values were represented using the normalization techniques provided by each algorithm: *Fragments per Kilobase of Mapped reads* (FPKM), *Transcripts per Million* (TPM), *Trimmed Mean of M values* (TMM from *edgeR*), *Relative Log Expression* (RLE from *DESeq2*), *upper quartile* (UQ), *coverage* (Cov), *estimated counts* (Est_Counts) and *effective counts* (Eff_Counts). Gene level expression values from *RSEM* and *eXpress* were obtained by summing the transcript-level estimates. Details about these counting and normalization methods are given in Supplementary Note S1.

#### Pseudoalignment

These methods do not consider the classical alignment process and carry out alignment, counting and normalization in one single step. We applied three commonly used pseudoaligners: *Kallisto*^26^ (v.0.43.1), *Sailfish*^28^ (v.0.9.2) and *Salmon*^72^ (v.0.8.2). *Salmon* was executed using the FMD and the quasi-mapping-based (QMB) indexing modes. Details about pseudoaligners are given in Supplementary Note S1.

#### Statistical approaches for pipeline precision analysis

Precision was tested on the six DMSO treated control samples (CLA-T0 and CLB-T0) considering each cell line separately (three replicates by cell line). For this purpose, we considered the 107 HKg expressed in the six samples and in 32 healthy tissues previously reported (Supplementary Table S2). The median rank of the precision was calculated from the CoV of the 107 genes. The CoV is defined as:

$$ CoV = \frac{MAD}{{Median}} $$

where MAD is the median absolute deviation for each gene, given by:

$$ MAD = median\left( {\left| {X_{i} - median \left( X \right)} \right|} \right) $$

being X the expression value of the X gene and i the ith sample.

The choice of this parameter was justified because it is a non-parametric method that quantifies precision independently of the measurement units, and it also considers the median variability through the MAD parameter, but in the same context as the median. Given this background, we first calculated the CoV value for every single gene inside of each pipeline and each cell line. Secondly, we ranked CoV values in each of the 192 pipelines generated from the combination of all the above algorithms and methods (Fig. 1). Thirdly, we computed the median of the ranks for the 107 HKg in each pipeline and this value was considered as the precision index of each pipeline. The lower the index, the more precise the pipeline was. This procedure is graphically described in the upper panel of Fig. 2.

#### Statistical approaches for pipeline accuracy analysis

Accuracy was tested on the six DMSO treated samples considering separately each cell line. We calculated the accuracy of each pipeline based on the 32 gene expression values from qRT-PCR, which is considered as a gold standard method due to its accuracy and sensitivity^73^. The Pearson correlation coefficient (*r*) was used to assess the association between the RNA-seq data and the qRT-PCR Ct values. Then, we calculated a rank for the correlation coefficient of each gene along each pipeline. Finally, a median rank for each gene was calculated. The lower the rank, the more accurate the pipeline was. This procedure is graphically described in the bottom panel of Fig. 2. All *r* correlation coefficients were calculated using the corr.test function implemented in the *psych*^74^ R package (v.1.9.12.31).

#### TOP pipeline selection

The best pipelines were settled considering the summation of the precision and accuracy ranks inside each cell line, thus giving the same weight for both parameters. The lower the summation, the better the performance of the pipeline was. All the comparisons involving the pipeline ranks were statistically evaluated using the Kruskal–Wallis test followed by the Dunn’s post-hoc test. *p*-values were adjusted for multiple testing by the Benjamini–Hochberg False Discovery Rate (FDR) procedure^75^. All these calculations were made using the *dunn.test* package.

#### Differential gene expression methods for RNA-seq

**Figure 3:**
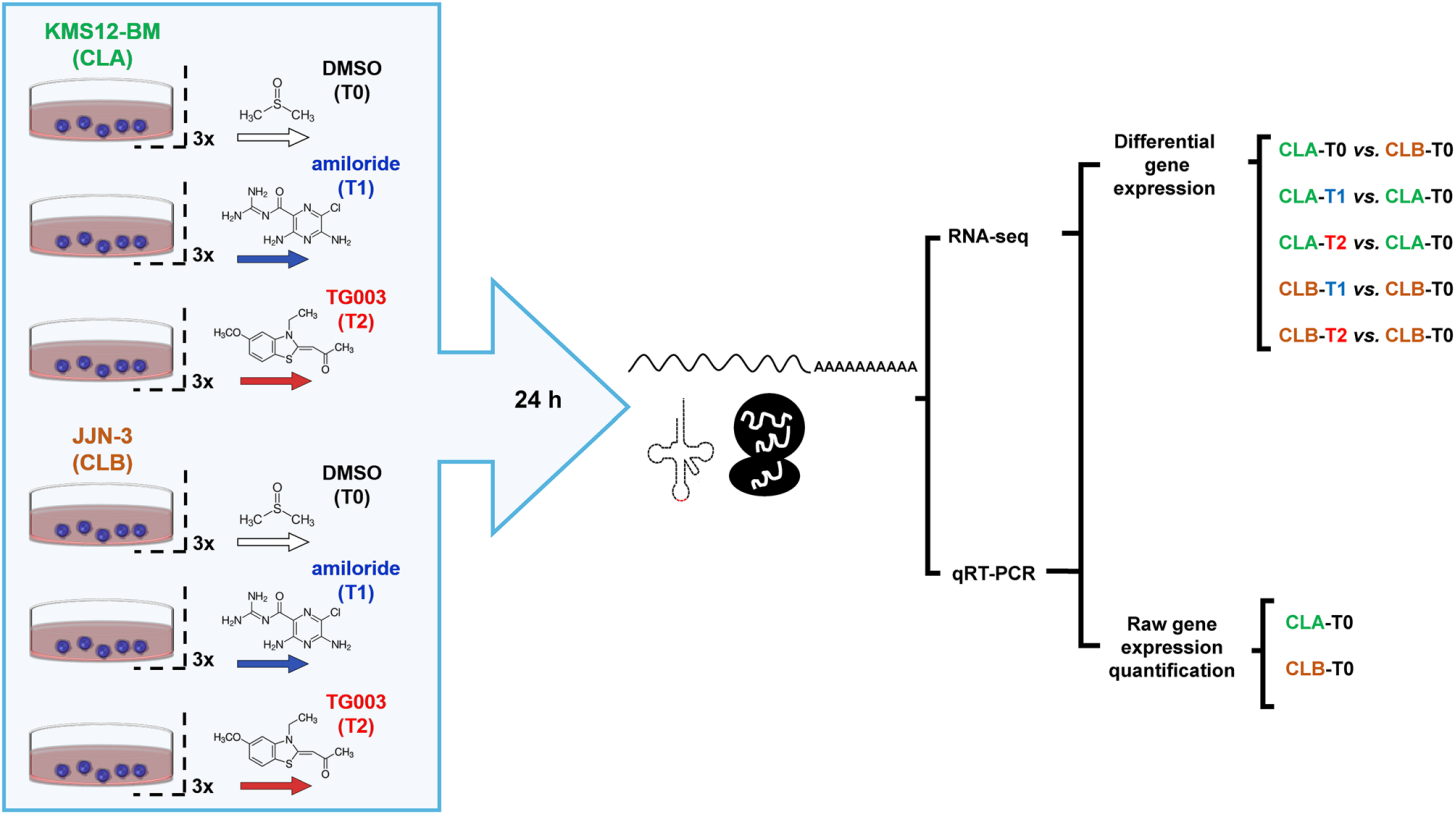
Experimental procedure. Two multiple myeloma cell lines (KMS12-BM [CLA] and JJN-3 [CLB]), two drugs (Amiloride [T1] and TG003 [T2]), and dimethyl-sulfoxide (DMSO) (treatment 0 [T0]) were used to conduct the RNA-seq and the qRT-PCR experiments. Control samples were used to carry out the raw gene expression quantification study, whilst all the 18 samples were used to perform the differential gene expression analysis.

Seventeen DE methods were evaluated, as the result of the combination of 11 DE algorithms and their different normalization options. Such methods can be divided into three categories: (a) methods that assume a negative binomial distribution of data: *baySeq*^76^ (v.2.10.0), *Cuffdiff*^77^ (v.2.2.1), *DESeq2*^78^ (v.1.16.1), *EBseq*^79^ (v.1.16.0) and the *edgeR*^80^ (v.3.18.1) generalized linear model (GLM), and exact test variants; (b) methods that assume a log-normal distribution, like *Ballgown*^81^ (v.2.8.4) and the *Trend* and *Voom limma*^82^ variants (v.3.32.10); and (c) non-parametric methods such as *NOISeq*^48^ (v.2.20.0) and *SAMseq*^83^ (available in the *samr* R package, v.2.0). Details about these methods are given in Supplementary Note S1. These DE methods were executed using the top 10 pipelines regardless of the normalization step, because most of DE methods only allow raw data as input. All these algorithms were assessed under five experimental contrasts, as the result of the combination of the two cell lines with the two treatment options and DMSO (Fig. 3). Contrasts were classified based on the number of DEGs detected. The sequence in descendent order of DEGs was: CLA-T0 *vs.* CLB-T0 > CLA-T1 *vs.* CLA-T0 > CLA-T2 *vs.* CLA-T0 > CLB-T1 *vs.* CLB-T0 > CLB-T2 *vs.* CLB-T0. All the above DEGs comparisons were carried out at 3 FDR cut-offs: FDR < 0.05, FDR < 0.01 and FDR < 0.001.

The similarity among the 17 methods was calculated in R through the *dist* function from the *stats* package (v.3.5.1) using the Euclidean distance as distance measure and group average as linkage method. Dendrograms were depicted using the *dendextend* package (v.1.13.3). The performance of the DE methods was determined through the measurement of 7 diagnostic test parameters: sensitivity or true positive rate (TPR), specificity or true negative rate (TNR), the positive predictive value (PPV), the negative predictive value (NPC), accuracy (ACC), the area under the receiver operating characteristic curve (AUC) and the Matthews correlation coefficient (MCC). These parameters were calculated using as reference the qRT-PCR Benjamini-Hockberg’s adjusted *p*-values in a two-sample *t*-test. The true positive, true negative, false positive and false negative estimates were defined based on these qRT-PCR adjusted *p*-values and compared with the adjusted *p*-values obtained by each RNA-seq DE method.

### Equipment

All the computational procedures for raw gene expression quantification were performed in a 64-bit computer with 264 GB of RAM and 64 CPUs installed with a Linux system CentOS 6.9. The DGE quantification was carried out in a 64-bit computer with 64 GB of RAM and 24 CPUs with either a Linux system Ubuntu 14.04 or a Windows 10 system.

## Results

### RNA-seq workflow

We tested the precision and accuracy of 192 RNA-seq pipelines in two independent and well-characterized MM cell lines at raw gene expression quantification level (RGEQ) (Fig. 3). These 192 pipelines are the result of the combination of different algorithms for trimming, alignment, counting and normalization (Fig. 1, left panel). Comparisons were made using non-parametric statistics based on ranks and median values as presented schematically in Fig. 2. We also tested 11 algorithms for DE combined with the normalization procedures available in their respective packages, which gave a total of 17 approaches (Fig. 1, right panel).

### Precision and accuracy of the evaluated pipelines

Precision was calculated using the 107 house-keeping reference genes (HKg) (Supplementary Table S2) individually for each cell line, as described in “Methods” and in the upper panel of Fig. 2. This analysis showed that pipelines using pseudoalignment algorithms were disproportionately represented among the top-ranked positions. Specifically, the most precise algorithm was *Salmon* in conjunction with TPM normalization. The least precise pipelines were those in which the normalization process was less strict, such as the *raw reads*, *Numreads*, *effective counts* and *estimated counts* (Supplementary Table S4).

The accuracy of the 192 pipelines was estimated by testing by qRT-PCR 32 of the 107 genes used for the precision analysis (highlighted genes in Supplementary Table S2). Accuracy was calculated based on the qRT-PCR results as described in the “Methods” section and in the bottom panel of Fig. 2. The top-ranked pipelines were those in which *HTSeq* was the counting method and TMM was the normalization approach. It was also noticed that the traditional alignment methods like *RUM* were situated in the top positions. The most accurate pipeline used the *Sailfish* pseudoaligner. However, most pipelines that used a pseudoalignment method were ranked in the lowest positions in the list for accuracy (Supplementary Table S5).

Finally, we generated a global ranking for all the tested pipelines, simultaneously considering the precision and accuracy on RGEQ. The top 10 ranked pipelines are shown in Table 1, and the complete ordered list is presented in Supplementary Table S6. Pipelines that used the counting algorithm *HTSeq* with its *Union* variant and the TMM normalization were the most represented in the highest positions of the pipeline ranking. We also observed that the *RUM, Tophat2* and *STAR* alignment algorithms appeared very frequently at the top positions of this list. We found no pattern for the trimming algorithms. These results clearly show that counting and normalization methods are the most critical steps in the RNA-seq analysis process. Particularly, considering the above results, we concluded that the combination of *Trimmomatic* + *RUM* + *HTSeq Union* + *TMM* was the most precise and accurate pipeline.

**Table 1:** Top 10 pipelines based on the overall precision and accuracy ranking.

Ranking	Trimming method	Alignment method	Counting method	Normalization method	Median precision	Median accuracy	Median overall precision and accuracy
1	Trimmomatic	RUM	HTSeq UNION	TMM	68.5	56	249
2	Trimmomatic	RUM	HTSeq INTER	TMM	68.5	57.75	252.5
3	BBDuk	RUM	HTSeq UNION	TMM	70	56.5	253
4	BBDuk	STAR	HTSeq UNION	TMM	68	62	260
5	Cutadapt	TopHat2	HTSeq UNION	TMM	62.5	67.75	260.5
6	BBDuk	TopHat2	HTSeq UNION	TMM	63.5	68	263
7	BBDuk	HiSat2	HTSeq UNION	TMM	63.5	69.25	265.5
8	BBDuk	TopHat2	HTSeq INTER	TMM	62.5	70.5	266
9	Trimmomatic	STAR	HTSeq UNION	TMM	69	64.75	267.5
10	Trimmomatic	STAR	HTSeq INTER	TMM	63.5	71	269

The lower the median overall precision and accuracy the better the pipeline performance was.

### Influence of individual RNA-seq analysis steps on raw gene expression quantification

We assessed the impact of each individual RNA-seq analysis step on the RGEQ. Thus, we performed this analysis at five levels: trimming, alignment, counting and normalization, and the pseudoalignment.

#### Trimming

**Figure 4:**
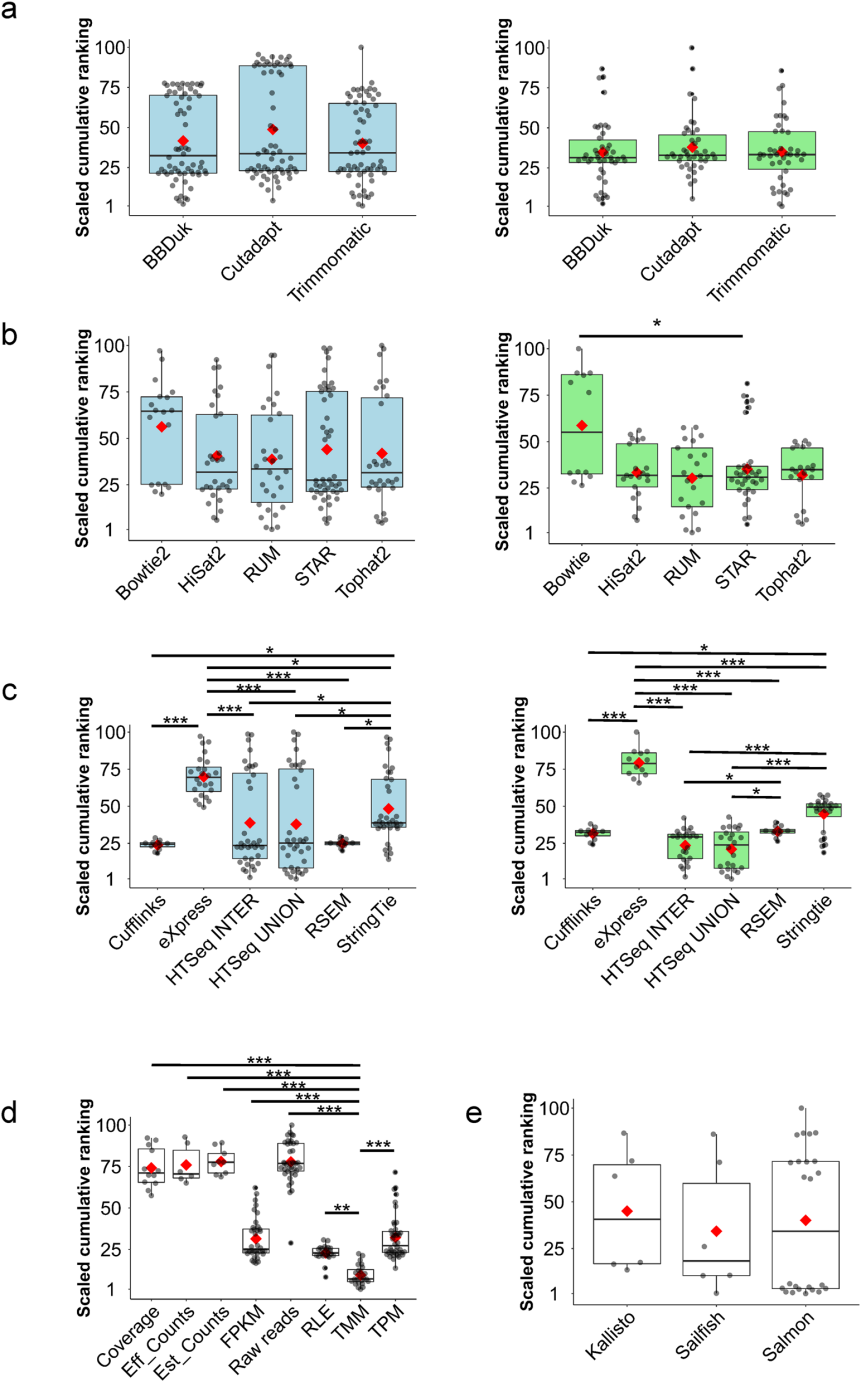
Influence of the algorithms on the RNA-seq raw gene expression quantification. Box-plot analysis of the 192 pipelines grouped by the algorithms used at each step of the procedure: (**a**) trimming algorithms, (**b**) alignment algorithms (**c**) counting methods, (**d**) normalization methods and (**e**) pseudoalignment algorithms. Coloured boxplots represent the scaled values (between 1 and 100) of the summation of the precision and accuracy ranking of the 192 pipelines before (blue) and after (green) the removal of pipelines that used raw reads, effective counts, estimated counts and coverage, which produced a bimodal data distribution. The red diamond represents the mean of the ranking reached by the pipelines that use the respective method or algorithm. The asterisks indicate the significance of the post-hoc Dunn’s test: *p < 0.05, **p < 0.01 and ***p < 0.001. Comparisons without asterisk are statistically insignificant (*p* > 0.05). Asterisks in (**d**) correspond to the *p*-values of the most significant method (TMM) against the other methods.

With respect to the effect of trimming, we explored the influence of three algorithms on RGEQ. We found that the three algorithms displayed different patterns regarding the surviving paired reads and the alignment rate. Thus, *Cutadapt* was the algorithm that produced the highest overall rate of surviving paired reads (95.5%) and the lowest alignment rate (93.4%). On the other hand, *BBDuk* obtained the highest percentage of aligned reads (97.5%) (Supplementary Table S7). Despite these divergences, we did not find statistically significant differences in RGEQ among the three algorithms (FDR > 0.05) (Fig. 4a and Supplementary Table S8).

#### Alignment

At this level, we evaluated five aligners that represent three alignment methodologies: to align against the reference genome, to align against the reference transcriptome and a hybrid approach. This process was evaluated on 156 pipelines because the 36 remaining pipelines were used to assess the pseudoalignment performance. There were two parameters that could affected the RGEQ ranking at this level: the number of concordant alignments and the unmapped reads output by each aligner. We found huge differences for these two parameters among the five aligners. *STAR* was the algorithm with a higher number of concordant alignments (median = 93%, MAD = 1.0), closely followed by *TopHat2* (Median = 90.1%, MAD = 2.0). On the other hand, *Bowtie2* only reached a 41.5% (MAD = 1.7) of concordant alignments, showing statistically significant differences with the other four aligners (FDR < 0.05) (Supplementary Fig. S2). Regarding to the unmapped reads, *HiSat2*, *and STAR* outperformed the other aligners (FDR < 0.05), both producing less than 5% of unmapped reads, although *HiSat2* was particularly effective with only 1.6% (MAD = 0.3) unmapped reads (Supplementary Fig. S2).

In view of this, we tested the influence of these aligners on the RGEQ and, despite their particularities, we did not find any statistically significant differences (FDR > 0.05) between the median ranks of the pipelines involving these alignment algorithms (Fig. 4b and Supplementary Table S9). However, we found a bimodal distribution in the RGEQ ranking values for these algorithms. We then investigated the cause of this bimodal distribution and we discovered that it was associated with the method employed for normalization. Therefore, the pipelines that used raw reads, effective counts, estimated counts and coverage, reached the lowest ranks. A reanalysis removing pipelines with these normalization approaches revealed that *Bowtie2* pipelines reached poorer ranks of RGEQ than pipelines that employed other alignment methods (Fig. 4b).

#### Counting and normalization

After the alignment step, we evaluated the influence of the counting methods on RGEQ. Pipelines based on *Cufflinks* and *RSEM* reached the highest positions in the pipeline ranking followed by *HTSeq* and *Stringtie* based pipelines. These last two methods also obtained high-ranking positions, but they showed a bimodal distribution in their rank values that was eventually translated in a greater variability, similarly to that observed in the alignment step (Fig. 4c). The removal of the pipelines that used raw reads, effective counts, estimated counts and coverage as normalization approach, caused the clearance of the bimodal distributions and revealed a better performance of *HTSeq* pipelines (*HTSeq-Inter* and *HTSeq-Union*, Fig. 4c). It also should be noted that the *eXpres*s pipelines showed the poorest rates for the sum of precision and accuracy (Fig. 4c and Supplementary Table S10).

Next, we evaluated the influence of normalization methods after the counting procedure. Pipelines using the *Trimmed Mean of M values* (TMM) method performed the best on RGEQ. Other normalization methods, such as *Relative Log Expression* (RLE) that was second best, and *Transcript Per Million* (TPM) or *Fragments Per Kilobase of Mapped reads* (FPKM), also reached high-ranking positions for precision and accuracy in RGEQ analysis (Fig. 4d and Supplementary Table S11). Considering these results, the normalization procedure proved to be an essential step in RGEQ, since we detected the higher statistically significant differences among the pipeline ranks based on the normalization method used.

#### Quantification by pseudoalignment

The RGEQ analysis was completed with the evaluation of three pseudoalignment algorithms considering their different alignment modes. These algorithms have a major advantage over traditional alignment methods as they carry out alignment and counting in a single step. Regarding the performance of these algorithms on RGEQ, we found no statistically significant differences (FDR > 0.05) between their cumulative ranks of precision and accuracy (Fig. 4e and Supplementary Table S12). When we compared these pseudoalignment algorithms with traditional aligners, we found a similar performance on RGEQ (FDR > 0.05) among the algorithms tested from both methodologies (Supplementary Table S13).

### Performance of differential gene expression methods

We tested 17 DE methods obtained from the combination of the different DE and normalization approaches. They were tested under six experimental conditions with three biological replicates per condition. Comparisons under these conditions are explained in Fig. 3. The efficiency of the DE procedures was checked at three commonly used FDR cut-offs: FDR < 0.05, FDR < 0.01 and FDR < 0.001.

**Figure 5:**
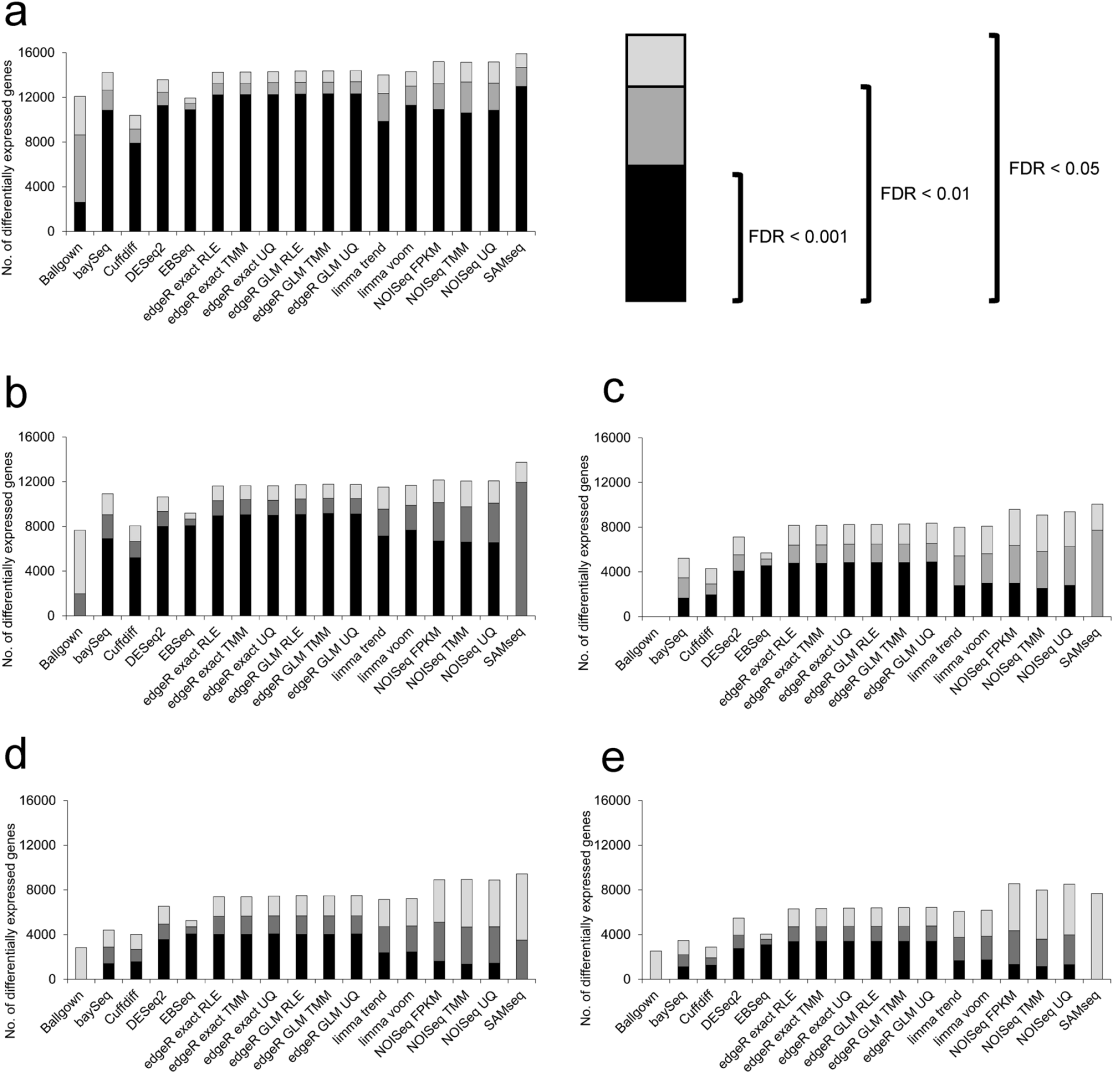
Differential expression detection. Number of differentially expressed genes (DEGs) detected by the 17 methods of differential expression at three FDR cut-offs: 0.05, 0.01 and 0.001. Panels represent different group comparisons in descending order based on the number of DEGs. (**a**) KMS12-BM (CLA) + DMSO (T0) *vs.* JJN-3 (CLB) + DMSO (T0) (**b**) KMS12-BM (CLA) + Amiloride (T1) *vs.* KMS12-BM (CLA) + DMSO (T0) (**c**) KMS12-BM (CLA) + TG003 (T2) *vs.* KMS12-BM (CLA) + DMSO (T0) (**d**) JJN-3 (CLB) + Amiloride (T1) *vs.* JJN-3 (CLB) + DMSO (T0) (**e**) JJN-3 (CLB) + TG003 (T2) *vs.* JJN-3 (CLB) + DMSO (T0).

First, we compared the detection ability among the DE methods. There was great homogeneity among the methods tested in the comparisons with a large number of gene expression changes. However, this homogeneity decreased as the compared experimental conditions became more similar (Fig. 5). Consistent with the findings of Seyednasrollah et al.^43^, we discovered that *Cuffdiff* was generally the algorithm that detected the smallest number of gene expression changes, whilst *SAMseq* was the method that detected the largest number of changes in all compared conditions. It was also observed that *SAMseq* itself and *Ballgown* lost detection power at FDR < 0.01 and FDR < 0.001 levels. *BaySeq*, *EBSeq* and *Cuffdiff* also lost detection power when the contrasted conditions were more similar. On the other hand, methods derived from *edgeR*, *limma* and *NOISeq* were stable in all comparisons at the three FDR levels analysed (Fig. 5a–e). The similarity analysis through the Euclidean distance among these 17 methods detected a high correspondence among *edgeR*, *limma* and *DESeq2* in all scenarios. However, methods such as *SAMseq*, *Cuffdiff, EBSeq* and *Ballgown* showed greater distances than most of the other methods. *NOISeq,* meanwhile, was the most variable method regarding the DEG scenario (Supplementary Fig. S3).

**Figure 6:**
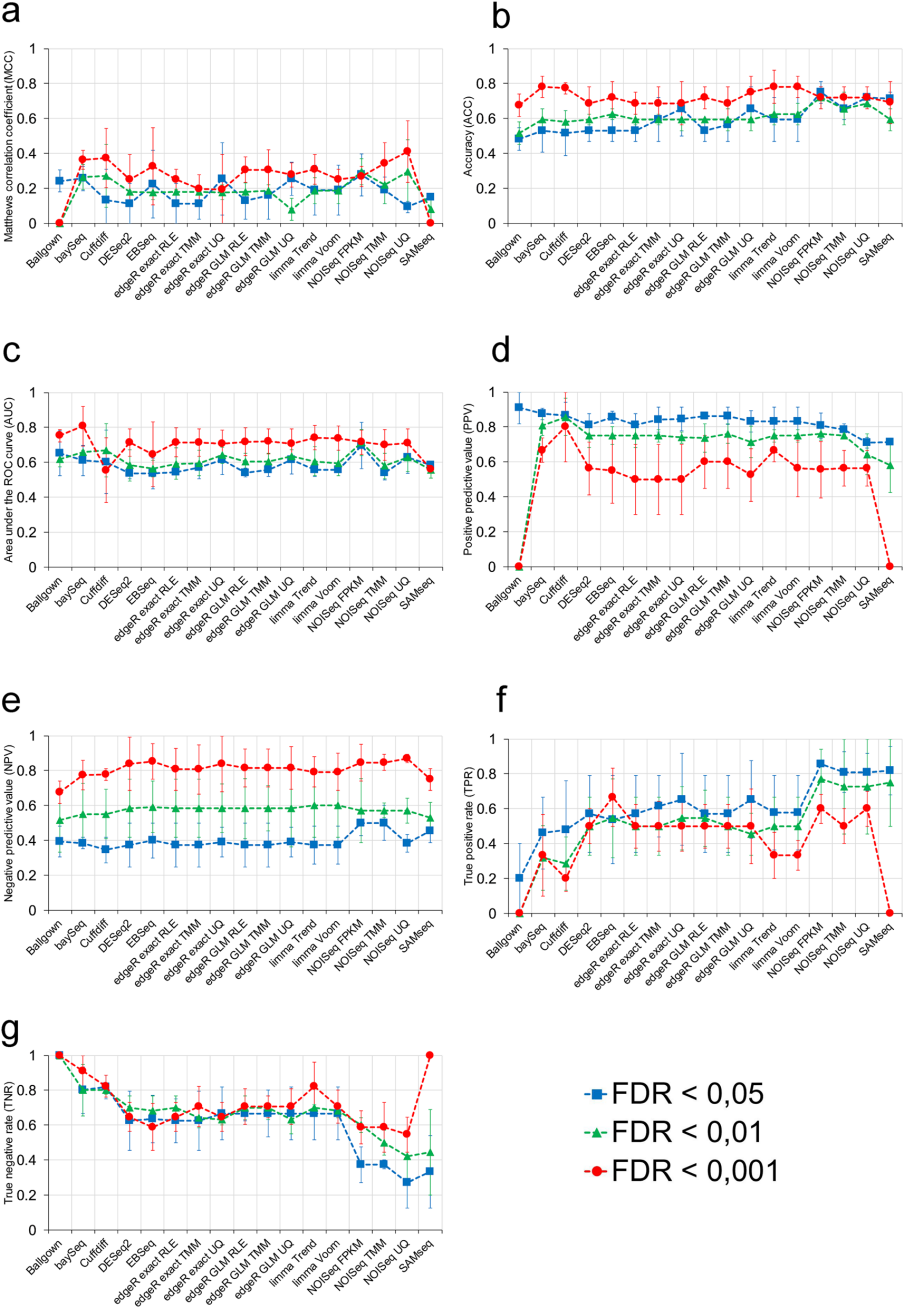
Analysis of performance of the 17 differential gene expression methods through the measurement of 7 diagnostic test parameters. (**a**) Matthews correlation coefficient (MCC), (**b**) accuracy (ACC), (**c**) area under the ROC curve (AUC), (**d**) positive predictive value (PPV), (**e**) negative predictive value, (**f**) true positive rate (TPR), and (**g**) true negative rate (TNR). Performance was measured at three FDR cut-off levels: FDR < 0.05, FDR < 0.01 and FDR < 0.001 for the 17 methods.

We also analysed 7 diagnostic test parameters for each DE method using qRT-PCR as described in the “Methods” section. The individual analysis of each parameter revealed substantial differences among the 17 methods (Fig. 6). Particularly, regarding the MCC (Fig. 6a), which evaluates the quality of the classification achieved by each method, some DE methods such as *NOISeq*, *Cuffdiff*, *baySeq* and some of the *edgeR* variants showed a moderate and positive relationship (MCC > 0.3) between their results and the true model. Concerning ACC and AUC (Fig. 6b,c), the most accurate methods, that is, those with a higher ACC, were *limma trend*, *limma voom* and *baySeq* at FDR < 0.001 (ACC = 0.78), while the method with a greater AUC, or in other words, the method with the highest discrimination capacity, was *baySeq* at FDR < 0.001 (AUC = 0.81).

Next, we carried out a performance analysis considering simultaneously 7 parameters in three experimental approaches: performance by number of DEG scenario, performance by statistical significance cut-off and overall performance. Regarding the performance by the number of DEGs, we observed great variability among the 17 DE methods, so that the first ranking position was reached by different methods in the five analysed scenarios. In this way, methods such as *EBseq*, *DESeq2* or *baySeq* showed a highly variable behaviour depending on the number of DEGs of the analysed scenario (Supplementary Fig. S4). On the other hand, the analysis of statistical significance revealed some methods with fairly high and stable performance ranks: *limma trend*, *limma voom* and *baySeq* (with also good ranks observed for *edgeR exact* and *NOISeq*, but suspected of greater variability depending on whether they used UQ, FPKM or TMM) (Supplementary Fig. S5).

To carry out the third approach, we considered both the five DEG scenarios and the three statistical significance levels. Taken together, *baySeq* and *NOISeq UQ* were the methods that showed a better behaviour even though both classified between the 9th and the 17th place in at least four of the approaches tested (Supplementary Fig. S6). These two methods were closely followed by *limma trend*, *limma voom*, and *edgeR GLM*.

**Figure 7:**
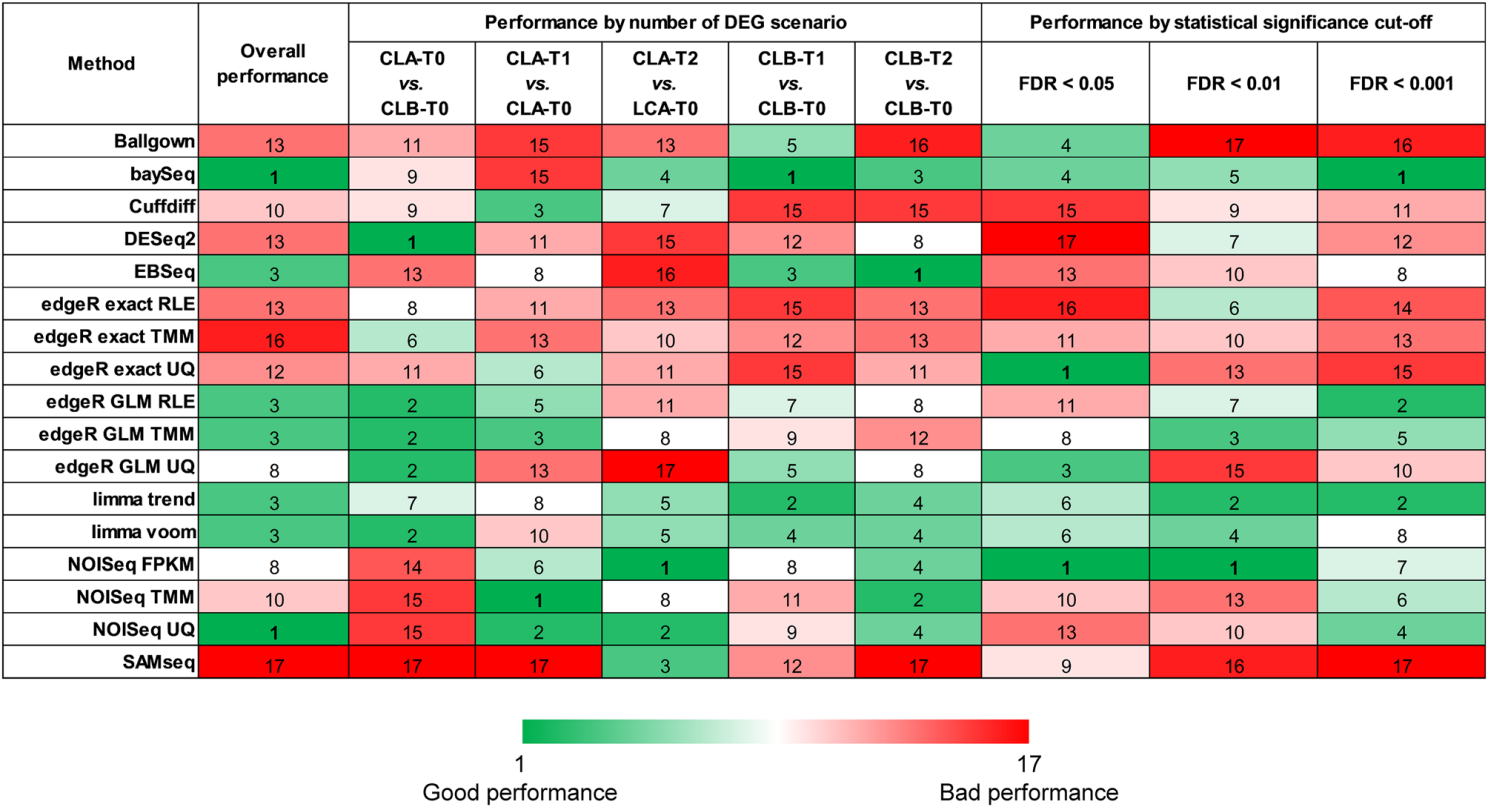
Summary of the performance of the RNA-seq gene differential expression analysis methods. This graph includes three experimental approaches for the 17 methods: performance by number of DEG scenario, performance by statistical significance cut-off and overall performance.

All these performance analyses are summarised jointly considering all the previous approaches in Fig. 7. We highlight that the most balanced method was *limma trend* since, unlike the other 16 methods, it was ranked among the 8 best methods in all the approaches. Other methods with good performance in all the scenarios were *baySeq*, *NOISeq FPKM*, *limma voom* and some variants of *edgeR GLM*.

To complete the DE analysis, we estimated the influence of normalization on DEGs detection by the DE methods that admitted multiple normalization approaches. We observed subtle differences in DEGs detection between the normalization approaches with regard to AUC and ACC, particularly in some analysis scenarios of the *NOISeq* algorithm, in which the FPKM normalization notably surpassed the other methods (Supplementary Fig. S7).

## Discussion

The findings of our study propose an optimal workflow for RNA-seq gene expression data analysis, based on the performance of multiple algorithms and pipelines freely available to the scientific community.

On a first stage, we performed a comparison of 192 pipelines to quantify raw gene expression. According to our results, the pipeline that obtained the best precision and accuracy rankings, and therefore it was the best combination of algorithms, was *Trimmomatic* + *RUM* + *HTSeq Union* + *TMM*. Despite this fact, the combination of methods that made the difference were *HTSeq* at the counting level and TMM at normalization level, as they were part of 10 out of the top 10 pipelines. On the other hand, the pipelines consistent on raw alignments, *Stringtie*’s coverage and *Salmon*’s *NumReads* occupied most of the last positions in the ranking, confirming the importance of a proper combination of counting and normalization methods.

In this work, we also compared the most commonly used methods over the stages of RNA-seq analysis: trimming, alignment, counting and normalization. Interestingly, we did not find substantial differences in RGEQ related to the trimming algorithm or the aligner used in the RNA-seq data analysis. This last finding is consistent with the recent work of Schaarschmidt et al. in *Arabidopsis thaliana* samples^84^, showing that the seven analysed aligners could be equally applied in RNA-seq data analysis. On the other hand, we found that the counting and normalization step was shown to be a critical step in our RNA-seq data analysis. This has also been reported by Robert et al.^85^, who demonstrated that the counting step may overestimate or underestimate the level of gene expression. We also detected a preponderance of the *HTSeq* counting method (under the *Union* and *Intersection-strict* variants) in the top-ranked positions in our pipeline list. With respect to data normalization, pipelines based on the TMM method outperformed those using RLE, FPKM, TPM and all the other normalization approaches tested. This finding is consistent with those of Wu et al.^21^ and Maza et al.^20^, but is not in agreement with those of Li et al.^22^, who concluded that gene normalization methods did not improve the gene expression calculations provided by the raw counts.

We also measured the performance of the pseudoalignment on RGEQ. Recently, pseudoaligners have entered the scene as an alternative to the classic alignment algorithms. One of their main advantages is that they can carry out the processes of alignment, counting and normalization in a single step. We have seen that pipelines based on pseudoaligners have a good precision in gene expression estimation, but their accuracy is inferior to that of the classic aligners. In any case, pseudoaligners could be a good alternative as RNA-seq data exploration tools since their execution time is faster than the conventional alignment methods.

Regarding RNA-seq DGE quantification, we established that the performance of the methods depended on the number of DEGs present under the contrasted experimental conditions and the FDR cut-off employed to determine statistical significance. Our systematic analysis revealed that *limma trend* obtained the best results in terms of performance, closely followed by *limma voom*, *NOISeq FPKM*, *baySeq*, and some derivations of *edgeR*. It is interesting that even though the assumptions about the underlying distribution differ, the performance of these methods is comparable, as was also described by Rapaport et al.^39^ with other sets of methods. Of note, in spite of their similar performance, we detected differences in behavioural patterns for these methods. Whilst *limma trend*, *limma voom* and *edgeR* showed a good balance among the seven diagnostic test parameters analysed, *baySeq* and *NOISeq* tended to be biased in favour of some of them. In the case of *baySeq*, we found that its performance was sustained by the excellent specificity of this method, however, we detected a substantial lack of sensitivity. With respect to *NOISeq*, its performance was supported by its good sensitivity and precision, but we found low levels of specificity. A similar pattern of unequal performance between diagnostic test parameters for *NOISeq* was also reported in the work of Williams et al.^51^, who concluded that *NOISeq* exhibited the lowest recall (sensitivity), but agreed with our work in the high precision of this algorithm.

Another important aspect of the DE analysis is the possible influence of normalization on the results. Normalization is a critical step in RNA-seq DGE analysis since it enables expression levels to be compared. According to our results, the choice of the normalization method had little effect on DEGs detection compared to RGEQ. These findings are supported by the research of Assefa et al.^86^ and Seyednasrollah et al.^43^, who reported a considerable overlap among DE methods regardless of the normalization approach used. However, our results are in contrast with those of Dillies et al.^25^, Bullard et al.^87^ and Zyprych-Walczak et al.^88^, possibly because these authors evaluated several normalization approaches regardless of the DE method, while in our study we only assessed the normalization provided by each DE method. It is also of note that *Ballgown* and *SAMseq* performed worse than others when the compared conditions had a low number of DEGs. It must be borne in mind that this bad behaviour may lead to a high frequency of false-negative deregulated genes in the data analysis.

## Supplementary information

Supplementary Information

Supplementary Information

## Data Availability

RNA-seq data used in this study are deposited in the Gene Expression Omnibus (GEO, https://www.ncbi.nlm.nih.gov/geo/) under the accession number GSE95077.
