# Supplementary material for: Systematic comparison and assessment of RNA-seq procedures for gene expression quantitative analysis
Source: Sci Rep. 2020 Nov 12;10:19737. doi: 10.1038/s41598-020-76881-x (PMC7665074; doi:10.1038/s41598-020-76881-x)
Supplement: Supplementary file 1 — Supplementary Information [file 41598_2020_76881_MOESM1_ESM.pdf]

Supplementary note

Systematic comparison and assessment of RNA-seq procedures for gene expression quantitative analysis

Luis A. Corchete[1-5*], Elizabeta A. Rojas[1-3], Diego Alonso-López[2], Javier De Las Rivas[2,3], Norma C. Gutiérrez[1-3,5] and Francisco J. Burguillo[4]

[1] Hematology Department, University Hospital, Salamanca, 37007, Spain.

[2] Cancer Research Institute-IBMCC (USAL-CSIC), Salamanca, 37007, Spain.

[3] Institute of Biomedical Research of Salamanca (IBSAL), Salamanca, 37007, Spain.

[4] Faculty of Pharmacy, University of Salamanca, Salamanca, 37007, Spain.

[5] Center for Biomedical Research in Network of Cancer (CIBERONC), Salamanca, Spain.

*Correspondence should be addressed to L.A.C. (lacorsan@usal.es).

Supplementary note

RNA-seq analysis, selected options

1. Trimming algorithms

A- *Trimmomatic* (v.0.35)

-jar trimmomatic-0.35.jar

PE # Paired-end mode

-threads 32

-phred33

ILLUMINACLIP:TruSeq3-PE.fa:2:30:10:7:true #fasta with paire-end adapter sequences : mismatches : palindrome clip threshold : simple clip threshold

LEADING:3 #Minimum base quality (leading region)

TRAILING:3 #Minimum base quality (trailing region)

SLIDINGWINDOW:4:20 #Number of bases in the window : minimum average quality of the window

MINLEN:51 #Minimum read length of read to be kept

B- *Cutadapt* (v.1.12)

cutadapt

-m 51 #Minimum read length of read to be kept

-j 32 #Number of cores

-a file:TruSeq3-PE.fa #Adapter sequences (forward)

-A file:TruSeq3-PE.fa #Adapter sequences (reverse)

-q 20 #Quality cutoff to trim end bases from reads

-e 0.06 #Maximum error rate

C- *BBDuk* (v.Oct.,23,2015)

ref=TruSeq3-PE.fa #Adapter sequences

ktrim=r #Trim kmers to the right

k=31 #kmer size

mink=11 #Look for shorter kmers with lengths from 31 to 11

hdist=2 #Hamming distance

rcomp=t #Look for kmers and their reverse complements

tbo # trim adapters based on pair overlap detection

tpe # trim both reads to the same length

qtrim=rl #Quality trim the right and the left sides

trimq=20 #Quality cutoff

minlength=51 #Minimum read length of read to be kept

2. Alignment algorithms

A- *Tophat2* (v.2.1.0),

<u>Tophat2 uses Bowtie2 genome indexes</u>

tophat2

--GTF Homo_sapiens.GRCh37.82.gtf

--library-type fr-firststrand

--mate-inner-dist (<u>adjust depending on the sample</u>)

--read-gap-length 2

--read-mismatches 2

--min-anchor-length 8

--num-threads 32

B- *STAR* (v.2.5.3a)

<u>Indexes were built through the following commands:</u>

.STAR

--runMode genomeGenerate

--runThreadN 32

--genomeDir ./star_indexes

--genomeFastaFiles ./genome.fa #Genome fasta

--sjdbGTFfile ./Homo_sapiens.GRCh37.82.gtf #Ensembl reference genes

--sjdbOverhang 100 #Length of the donor/acceptor sequence on each side of the junctions

<u>STAR options (genome based):</u>

STAR

--genomeDir ./star_indexes

--runThreadN 32

--readFilesType Fastx

--readMapNumber -1

--readStrand Unstranded

--clip3pNbases 0

--clip5pNbases 0

--outFileNamePrefix Sample_STAR

--outSAMtype BAM Unsorted

STAR options (transcriptome based):

STAR

--quantMode TranscriptomeSAM

--readFilesType Fastx

--readMapNumber -1

--readStrand Unstranded

--clip3pNbases 0

--clip5pNbases 0

--runThreadN 32

--outSAMtype BAM Unsorted

C- *Hisat2* (v.2.0.0)

Indexes were built through the following command:

hisat2-build

~/Homo_sapiens/genome.fa #Genome fasta

~/Homo_sapiens/genome.hisat #Output

Hisat2 options:

hisat2

-q #reads are fastq files

-p 32 #number of cores

-x ~/Homo_sapiens/genome.hisat

--rna-strandness RF

--known-splicesite-infile ~/Splicesites_GRCh37.82.txt

D- *Bowtie2* (v.2.2.6)

bowtie2

--fr #strand orientation

--minins 0 #The minimum fragment length for valid paired-end alignments

-x ~/Bowtie2Index/genome

-p 32 #Number of cores

E- *RUM* (v.2.0.5_06)

rum_runner align

--chunks 32 # Number of cores

--index-dir ~/rum_indexes/hg19

3. Counting methods

A- *Cufflinks* (v.2.2.1)

cufflinks

-p 32 # Number of cores

--library-type fr-firststrand

-G ~/Homo_sapiens.GRCh37.82.gtf #Ensembl gene reference

–max-mle-iterations 5000

B- *eXpress* (v.1.5.1)

express

--rf-stranded

~/ref/human_ensembl.transcripts.fa #Transcriptome reference

C- *HTseq* (v.0.6.1p1)

Intersection-strict option:

-m HTSeq.scripts.count

-m intersection-strict # HTseq mode

-s reverse #Whether the data is from a strand-specific assay

-r name #Alignment sorted by name

-a 10 #Minimum alignment quality value

-i gene_id # Attribute to be used as feature ID

-f bam # Input format

Homo_sapiens.GRCh37.82.gtf #Ensembl gene reference

Union option:

-m HTSeq.scripts.count

-m union # HTseq mode

-s reverse #Whether the data is from a strand-specific assay

-r name #Alignment sorted by name

-a 10 #Minimum alignment quality value

-i gene_id # Attribute to be used as feature ID

-f bam # Input format

Homo_sapiens.GRCh37.82.gtf #Ensembl gene reference

D- *RSEM* (v.1.2.31)

Preparing Reference Sequences:

rsem-prepare-reference

 --gtf Homo_sapiens.GRCh37.82.gtf

--bowtie2 \

 genome.fa # Genome reference fasta

ref/human_ensembl # Gene references for Ensembl

Validate BAM:

rsem-sam-validator

Calculating Expression Values Bowtie (from fastq file)

rsem-calculate-expression

--paired-end

--bowtie2

--estimate-rspd

--append-names \

--output-genome-bam

Calculating Expression Values (From transcriptome BAM)

rsem-calculate-expression

--bam

--alignments ~/Sample.toTranscriptome.bam

--estimate-rspd

--paired-end

E- *Stringtie* (v.1.3.3b)

stringtie

-v # Verbose mode

-p 32 #Number of cores

-G ~/Homo_sapiens.GRCh37.82.gtf # Gene reference from Ensembl

-f 0.01 #Minimum isoform abundance

4. Pseudoalingment algorithms

A- *Kallisto* (v.0.43.1)

Building an index:

kallisto index

-i TransGRCh37_82_Kallisto.idx

/Transcriptome_REF/TransGRCh37_82.fa #Transcriptome reference

Quantification using bootstrap for Sleuth (Differential expression):

kallisto quant

--fr-stranded

-b 100 #Number of bootstrap samples

B- *Sailfish* (v.0.9.2)

Building an index

sailfish index

-t /Transcriptome_REF/TransGRCh37_82.fa

-o TransGRCh37_82_Sailfish

-k 31 # Minimum match size

Quantify

sailfish quant

-l ISR #library type: Inward, Stranded, Read 1 comes from the reverse strand

C- *Salmon* (v.0.8.2)

Quasi-mapping-based model:

Building an index

salmon index

-t /Transcriptome_REF/TransGRCh37_82.fa

-i TransGRCh37_82_SalmonQuasi

--type quasi

-k 31 # Minimum match size

Quantify

salmon quant \

-i TransGRCh37_82_SalmonQuasi

-l ISR #library type: Inward, Stranded, Read 1 comes from the reverse strand

Lightweight-alignment (FMD-based) mode

Building an index

index -t /Transcriptome_REF/TransGRCh37_82.fa -i TransGRCh37_82_SalmonFMD --type fmd

Quantify

salmon quant

-i TransGRCh37_82_SalmonFMD

-l ISR #library type: Inward, Stranded, Read 1 comes from the reverse strand

5. Differential expression methods (R and Linux Scripts)

A- baySeq (v.2.10.0) (R)

```
library(baySeq)
##Define null cluster
if(require("parallel")) cl <- makeCluster(8) else cl <- NULL

##Load data
tabla <- read.table("Expression_Data.txt",sep="\t",header=T,row.names=1)
tabla <- as.matrix(tabla)
##3 samples in 2 groups
replicates <- c("Group1","Group1","Group1","Group2","Group2","Group2")
groups <- list(NDE=c(1,1,1,1,1,1),DE=c(1,1,1,2,2,2))

##Combine count data and models
CD <- new("countData",data=tabla,replicates=replicates,groups=groups)

##Inferre library size from data
libsizes(CD) <-getLibsizes(CD)

##Negative Binomial Approach
##Estimate parameters
##################quasi-likelihood estimation of priors#############################
CD1 <- getPriors.NB(CD,samplesize=10000,estimation="QL",cl=cl)
##Estimate proportions of DE counts
CD1 <- getLikelihoods(CD1,cl=cl,bootStraps=3,verbose=FALSE)
options("max.print"=1E9)
options("width"=10000)
sink("DE_baySeq_QL.txt")
print(topCounts(CD1,group="DE",FDR=1))
sink()
sink("NDE_baySeq_QL.txt")
```

```
print(topCounts(CD1,group="NDE",FDR=1))

sink()
```

B- Cuffdiff (v.2.2.1) (linux)

```
##Assemble transcripts for each sample

cufflinks -p 12 --library-type fr-firststrand -G Homo_sapiens.GRCh37.82.gtf -o Sample_cufflinks
/Sample.bam


##Create a file called assemblies.txt:

./Sample_1/transcripts.gtf

./Sample_2/transcripts.gtf

./Sample_N/transcripts.gtf


##Run cuffmerge to create a single transcriptome annotation

cuffmerge -g Homo_sapiens.GRCh37.82.gtf -s genome.fa -p 32 assemblies.txt


##CuffQuant

cuffquant -g /media/luis/Seagate_8Tb_1/3_Tesis_RNAseq/Homo_sapiens.GRCh37.82.gtf -b
genome.fa -p 12 --library-type fr-firststrand 141048.bam


##Differentially expressed genes and transcripts

cuffdiff -o Cuffdiff_out -b genome.fa -p 32 -L Treatment,Control --library-type fr-firststrand -b
genome.fa -u merged_asm/merged.gtf \

Treatment_1.cxb,Treatment_2.cxb,Treatment_N.cxb \

Control_1.cxb,Control_2.cxb,Control_N.cxb
```

C- DESeq2 (v.1.16.1) (R)

```
library(DESeq2)

##Load a matrix with the counts

sampleTable <- read.table("Expression_Data.txt",header=T,sep="\t",row.names=1)

##Load a phenotype matrix with the sample names in the first column and without headings

## in the second column put the sample group under the heading "condition"

sampleCondition <- read.table("Phenotype.txt",header=T,sep="\t",row.names=1)

##Create the DESeq object

dds <-
DESeqDataSetFromMatrix(countData=sampleTable,colData=sampleCondition,design=~condition
)
```

```
##Establish the factor order in the analysis
dds$condition <- factor(dds$condition,levels=c("Group1","Group2"))
##Launch the differential expression analysis
dds <- DESeq(dds)
res <- results(dds)
res
##Save the results
write.csv(as.data.frame(res),file="Results_DESeq2.csv")

##Order the results by the adjusted p-value
resOrdered <- res[order(res$padj),]
##summary of the results
summary(res)
##count significant genes at padj<=0.05
sum(res$padj<=0.05,na.rm=T)
##Decrease the alpha value (default = 0.1)
res05 <- results(dds,alpha=0.05)
sum(res05$padj<=0.05,na.rm=T)
##MAplot
plotMA(res,main="DESeq2",ylim=c(-2,2))
##Obtain the unshrunken log2 fold Changes and lfcMLE
resMLE <- results(dds,addMLE=T)
head(resMLE,4)
plotMA(resMLE,MLE=TRUE,main="unshrunken LFC",ylim=c(-2,2))
##Gene count graphs
plotCounts(dds,gene=which.min(res$padj),intgroup="condition")
```

D- EBseq (v.1.16.0) (R)

```
library(EBSeq)
tabla <- read.table("Expression_Data.txt",sep="\t",header=T,row.names=1)
tabla <- as.matrix(tabla)
Sizes=MedianNorm(tabla)

EBOut=EBTest(Data=tabla,Conditions=as.factor(rep(c("Group1","Group2"),each=3)),sizeFactors
=Sizes, maxround=5)
```

```
EBDERes=GetDEResults(EBOut, FDR=1,Threshold_FC=1,Threshold_FCRatio=0)
##Calculate FC
FC <- PostFC(EBOut, SmallNum = 0.01)
##Obtain probabilities
Expresion <- cbind(EBDERes$PPMat,EBDERes$Status,FC$PostFC,FC$RealFC)
options("max.print"=1E9)
options("width"=10000)
sink("EBSeq_Results.txt")
print(Expresion)
sink()
```

E- edgeR (v.3.18.1) (R)

```
tabla <- read.table("Expression_Data.txt",header=T,sep="\t",row.names=1)
library(edgeR)
##Create list and calculate library sizes
group <- c(1,1,1,2,2,2)
d <- DGEList(counts=tabla,group=factor(group))
d
##Normalization
dt <- calcNormFactors(d,method="TMM")
##Estimate dispersion
d1 <- estimateCommonDisp(dt, verbose=T)
d1 <- estimateTagwiseDisp(d1)

design.mat <- model.matrix(~ 0 + dt$samples$group)
colnames(design.mat) <- levels(dt$samples$group)
d2 <- estimateGLMCommonDisp(dt,design.mat)
d2 <- estimateGLMTrendedDisp(d2,design.mat, method="auto")
# You can change method to "auto", "bin.spline", "power", "spline", "bin.loess".
# The default is "auto" which chooses "bin.spline" when > 200 tags and "power" otherwise.
d2 <- estimateGLMTagwiseDisp(d2,design.mat)

##Compare groups (exact test)
et12 <- exactTest(d1, pair=c(1,2))
options("max.print"=1E9)
```

```
options("width"=10000)
sink("edgeR_ExactTest_TMM.txt")
print(topTags(et12, n=length(tabla[,1]), adjust.method="BH", sort.by="PValue", p.value=1))
sink()

##Compare groups GLM Log Likelihood Ratio
fit <- glmFit(d2,design.mat)
lrt12 <- glmLRT(fit,contrast=c(-1,1))
sink("edgeR_GLM_TMM.txt")
print(topTags(lrt12 , n=length(tabla[,1]), adjust.method="BH", sort.by="PValue", p.value=1))
sink()
```

F- Ballgown (v.2.8.4) (linux and R)

```
#1# Estimate transcript abundances and create table counts for Ballgown (1 for each sample)
stringtie -e -B -p 32 -G Homo_sapiens.GRCh37.82.gtf -o ./Sample.gtf Sample.bam



#2# Run Ballgown in R
library(ballgown)
library(RSkittleBrewer)
library(genefilter)
library(dplyr)
library(devtools)

#Load the phenotype data for the samples
pheno_data = read.csv("Phenotype.csv")
##Format:ids Treatment cov2

#Read in the expression data that were calculated by StringTie or Cufflinks
bg = ballgown(dataDir = "Ballgown", samplePattern = "Sample", pData=pheno_data)

#Filter to remove low-abundance genes. One common issue with RNA-seq data is that genes
often have very few or zero counts
bg_filt = subset(bg,"rowSums(gexpr(bg) >=4) >=1",genomesubset=TRUE)

#Identify genes that show statistically significant differences between groups
```

```
results_genes_F = stattest(bg_filt, feature="gene", covariate="Treatment", getFC=TRUE,
meas="FPKM")


#Write the results to a csv file that can be shared and distributed:
write.csv(results_genes_F, "Ballgown_FPKM_results_gexpr.csv",row.names=FALSE)


##Add HUGO gene symbol
indices <- match(results_genes_F$id, texpr(bg, 'all')$gene_id)
gene_names_for_result <- texpr(bg, 'all')$gene_name[indices]
results_genes_F <- data.frame(geneNames=gene_names_for_result, results_genes_F)
write.csv(results_genes_F, "Ballgown_FPKM_results.csv",row.names=FALSE)
```

G- limma (v.3.32.10) (R)

```
library(limma)
library(edgeR)
tabla <- read.table("Expression_Data.txt",sep="\t",header=T,row.names=1)
dge <- DGEList(counts=tabla)
##design
targets<-readTargets("Targets.txt")
Treat<-factor(targets$Treatment,levels=c("Group1","Group2"))
design<-model.matrix(~Treat)
##Normalizing
dge <- calcNormFactors(dge)
##Differential expression: Limma trend
#convert counts to logCPM
logCPM <- cpm(dge,log=TRUE,priot.count=3)
##Fit the model
fit <- lmFit(logCPM,design)
fit <- eBayes(fit,trend=TRUE)
options("max.print"=1E9)
options("width"=10000)
sink("limma-Trend_Results.txt")
print(topTable(fit,coef=ncol(design),p.value=1, number= length(tabla[,1])))
sink()


##Voom method
```

```
v <- voom(dge,design,plot=T)
fitv <- lmFit(v,design)
fitv <- eBayes(fitv)
sink("limma-VOOM_Results.txt")
print(topTable(fitv,coef=ncol(design),p.value=1, number= length(tabla[,1])))
sink()
```

H- NOIseq (v.2.20.0) (R)

```
library(NOISeq)
tabla <- read.table("b_BM-DMvJJ-DM_1.4.2.1_Filt.txt",sep="\t",header=T,row.names=1)
myfactors = data.frame(Condition = c(rep("JJ", 3), rep("BM",3)))
mydata <- readData(data = tabla, factors = myfactors)



##Differential expression
##Available normalization methods: norm = "rpkm", "uqua", "tmm2, "none"
mynoiseqbio = noiseqbio(mydata, k = 0.5, norm = "rpkm", nclust = 15, plot = FALSE,
factor="Condition", lc = 0, r = 50, adj = 1.5,
a0per = 0.9, random.seed = 12345, filter = 0, depth = NULL,
cv.cutoff = NULL, cpm = NULL)

options("max.print"=1E9)
options("width"=10000)
sink("NOISeq-RPKM_Results.txt")
print(mynoiseqbio@results[[1]])
sink()



##k=Counts equal to 0 are replaced by k. By default, k = 0.5
##norm = Normalization method. It can be one of "rpkm" (default), "uqua" (upper quartile),"tmm" (trimmed mean of M) or "n" (no normalization).
##r Number of permutations to generate noise distribution by resampling.
## adj Smoothing parameter for the Kernel Density Estimation of noise distribution. Higher values produce smoother curves.
```

##nclust Number of clusters for the K-means algorithm. Used when the number of replicates per condition is less than 5.

##a0per M and D values are corrected for the biological variability by being divided by S + a0, where S is the standard error of the corresponding statistic and a0 is determined by the value of a0per parameter. If a0per is NULL, a0 = 0. If a0per is a value between 0 and 1, a0 is the a0per percentile of S values for all features. If a0per = "B", a0 takes the highest value given by 100*max(S).

##filter If filter=0, no filtering is performed

I- SAMseq (available in the *samr* R package, v.2.0).

```
library(samr)

tabla <- read.table("Expression_Data.txt", header=T, sep="\t", row.names=1)

##Group1 Samples (control)

n1 <- 3

##Group2 (treatment)

n2 <- 3

tabla.cls <- rep(c(1,2),c(n1,n2))

sam.out <- SAMseq(tabla,tabla.cls,resp.type="Two class
unpaired",nperms=1000,random.seed=123456,genenames=rownames(tabla),fdr.output =1)

options("max.print"=1E9)

options("width"=10000)

sink("SAMseq_Results.txt")

print(sam.out$siggenes.table)

sink()
```
