# Supplementary material for: Systematic comparison and assessment of RNA-seq procedures for gene expression quantitative analysis
Source: Sci Rep. 2020 Nov 12;10:19737. doi: 10.1038/s41598-020-76881-x (PMC7665074; doi:10.1038/s41598-020-76881-x)
Supplement: Supplementary file 2 — Supplementary Information [file 41598_2020_76881_MOESM2_ESM.pdf]

Supplementary Figures and Tables

Systematic comparison and assessment of RNA-seq procedures for gene expression quantitative analysis

Luis A. Corchete[1-5*], Elizabeta A. Rojas[1-3], Diego Alonso-López[2], Javier De Las Rivas[2,3], Norma C. Gutiérrez[1-3,5] and Francisco J. Burguillo[4]

[1] Hematology Department, University Hospital, Salamanca, 37007, Spain.

[2] Cancer Research Institute-IBMCC (USAL-CSIC), Salamanca, 37007, Spain.

[3] Institute of Biomedical Research of Salamanca (IBSAL), Salamanca, 37007, Spain.

[4] Faculty of Pharmacy, University of Salamanca, Salamanca, 37007, Spain.

[5] Center for Biomedical Research in Network of Cancer (CIBERONC), Salamanca, Spain.

*Correspondence should be addressed to L.A.C. (lacorsan@usal.es).

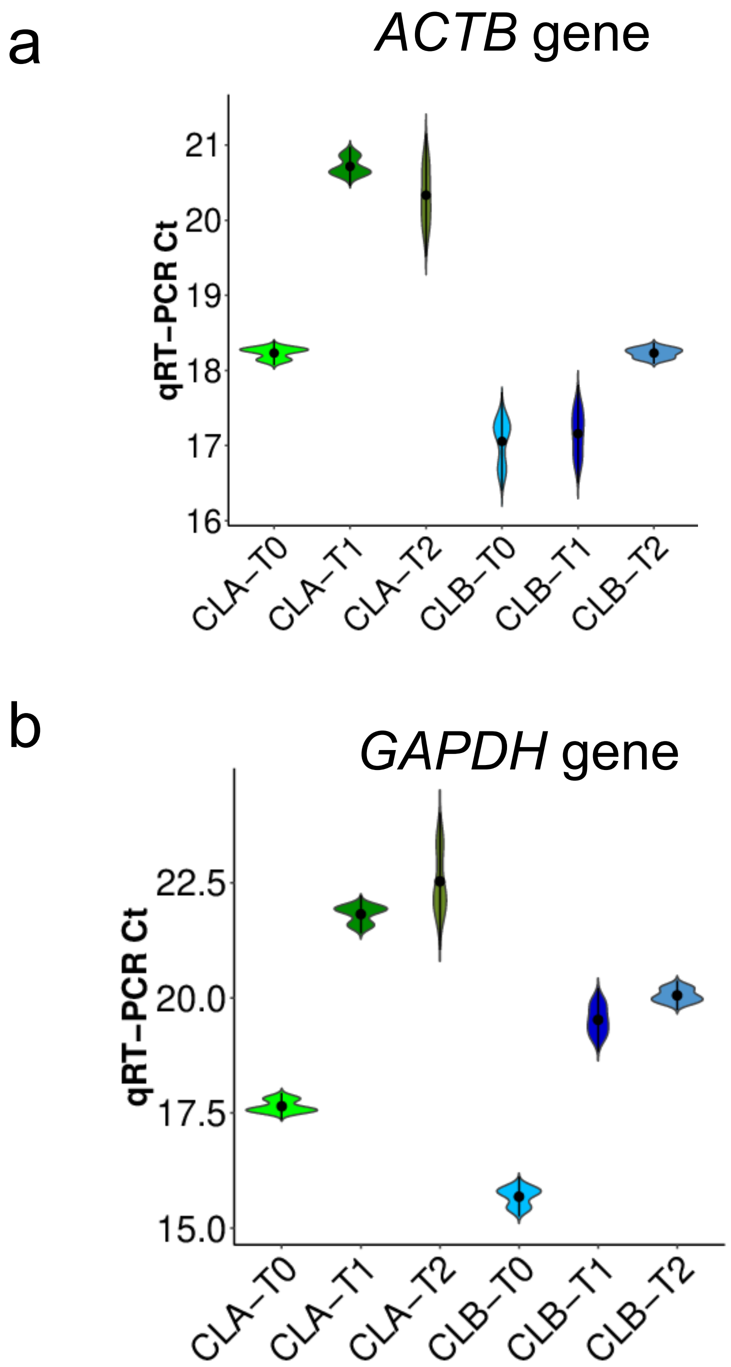

Supplementary Figure S1. qRT-PCR Ct values of the *GAPDH* and *ACTB* genes across all assayed conditions. Violin plots of (**a**) *ACTB* and (**b**) *GAPDH* genes using the Ct values from the qRT-PCR assay considering the KMS12-BM (CLA) and JJN-3 (CLB) cell lines, treated with Amiloride (T1) or TG003 (T2) and controls (T0).

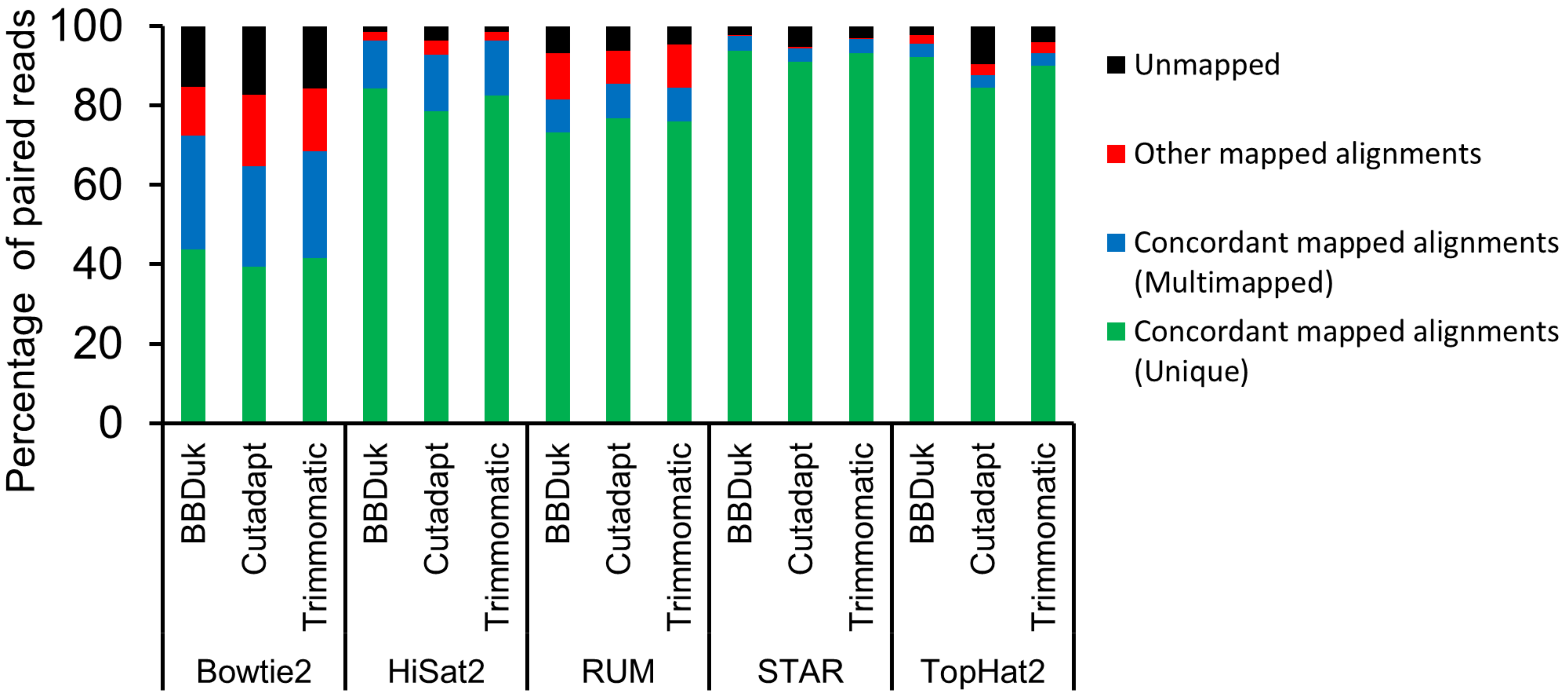

Supplementary Figure S2. Mapping summary of the alignment algorithms. The output of the five alignment methods was analysed considering the previous trimming algorithms (*BBDuk*, *Cutadapt* or *Trimmomatic*). The number of alignments are shown as percentages and are classified into four categories according the alignment type: unmapped alignments, concordant multimapped alignments, concordant uniquely mapped alignments and other alignments.

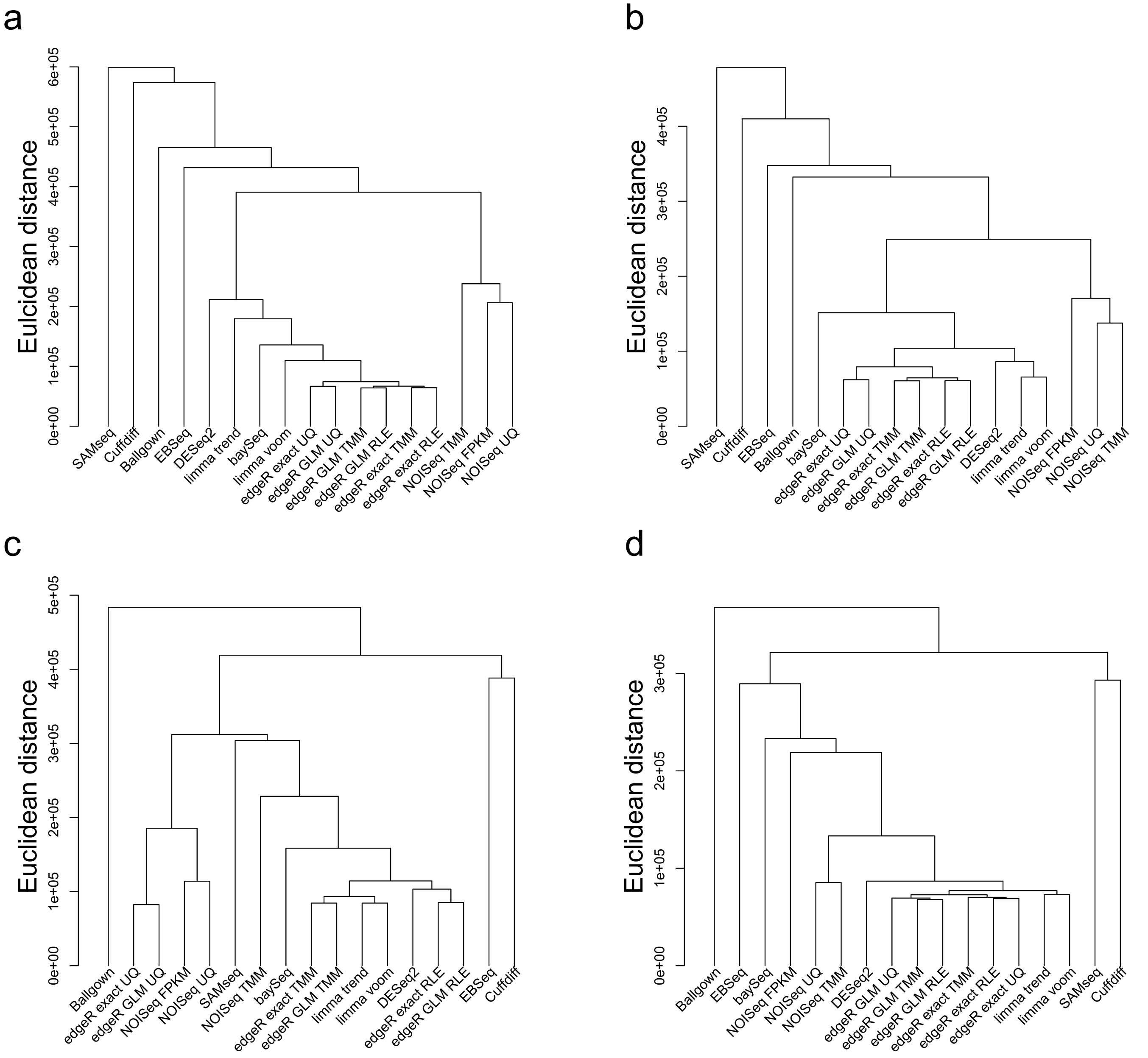

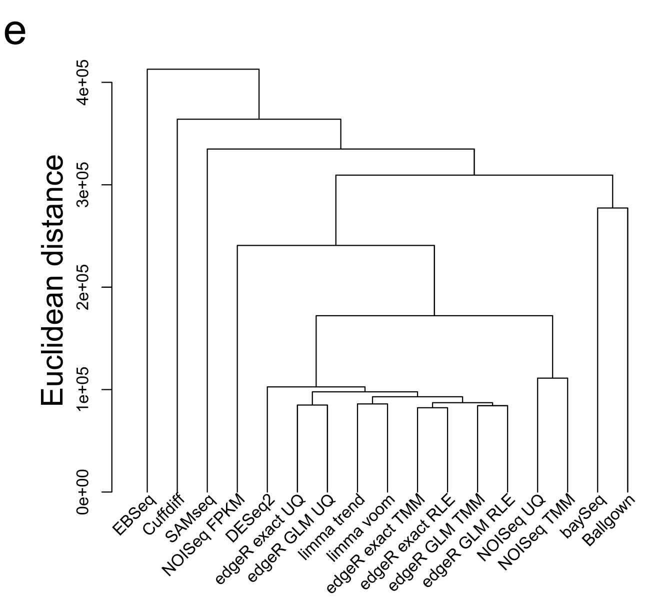

Supplementary Figure S3. Similarity among the 17 RNA-seq differential gene expression detection methods. Each graph represents the Euclidean distance among the 17 methods using dendrograms. (**a**) CLA-T0 vs. CLB-T0, (**b**) CLA-T1 vs. CLA-T0, (**c**) CLA-T2 vs. CLA-T0, (**e**) CLB-T1 vs. CLB-T0 and (**e**) CLB-T2 vs. CLB-T0. CLA = cell line A (KMS12MB); CLB = cell line B (JJN-3); T0 = DMSO; T1 = Amiloride; T2 = TG003

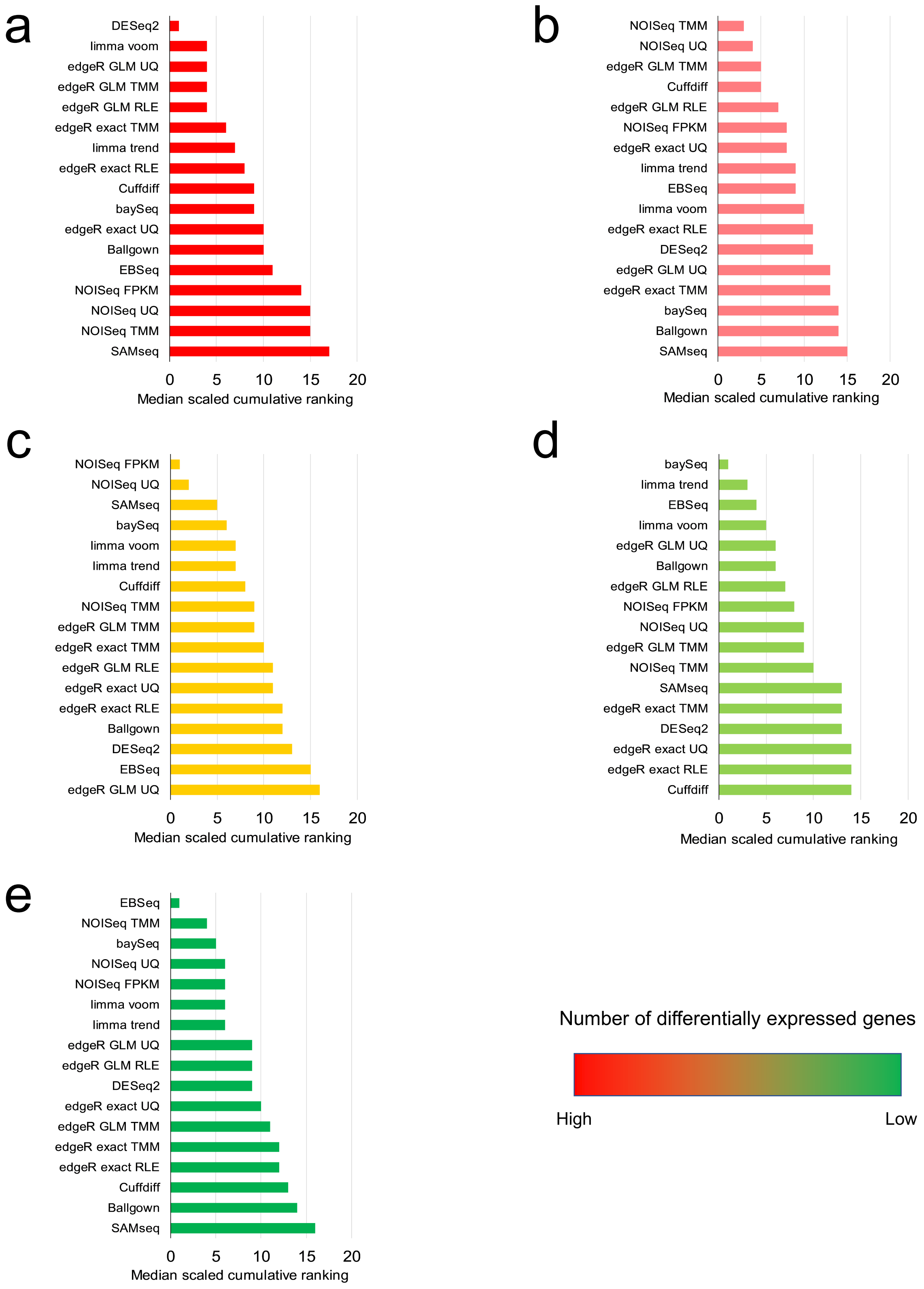

Supplementary Figure S4. Performance of the 17 differential expression methods based on the five contrasts proposed in this work. These five contrasts are defined according to the number of changes in gene expression between the contrasted conditions.(**a**) CLA-T0 vs. CLB-T0, (**b**) CLA-T1 vs. CLA-T0, (**c**) CLA-T2 vs. CLA-T0, (**d**) CLB-T1 vs. CLB-T0 and (**e**) CLB-T2 vs. CLB-T0, where CLA is the KMS12-BM cell line, CLB is the JJN-3 cell line, T0 is DMSO treatment, T1 is Amiloride treatment and T2 is TG003 treatment.

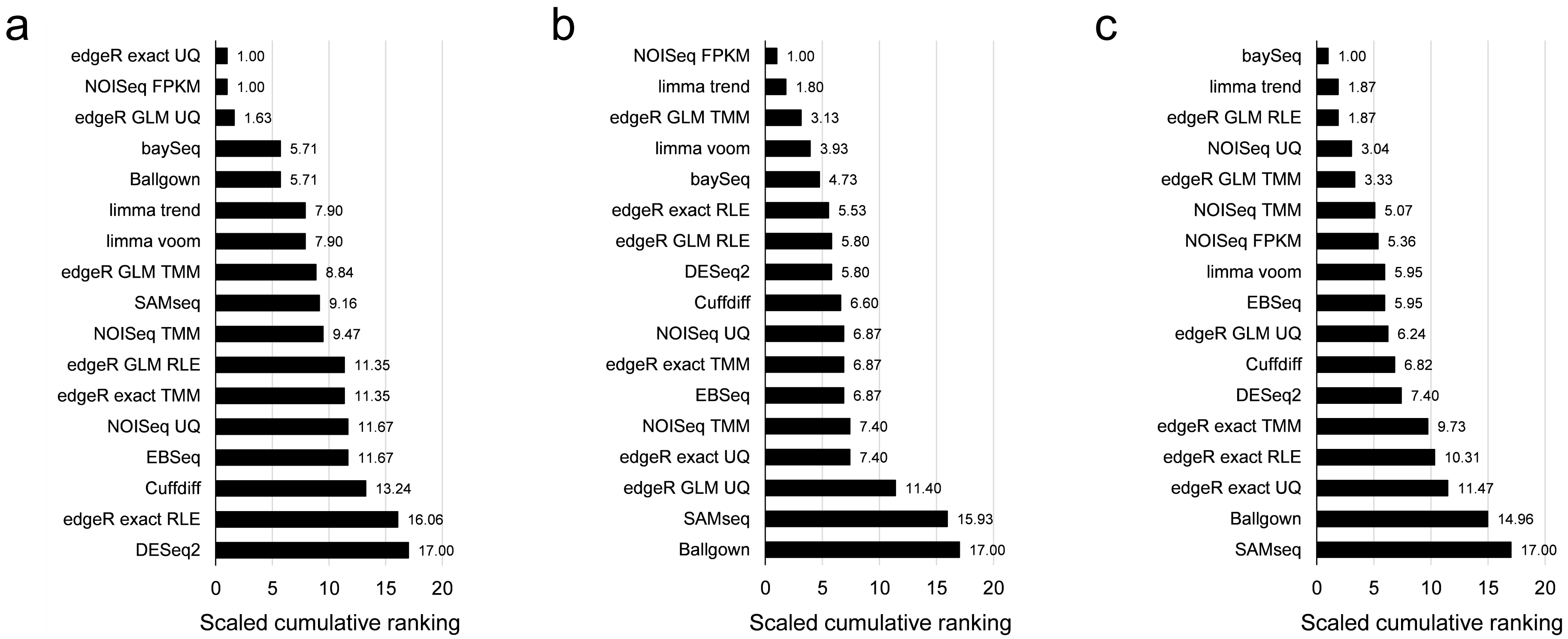

Supplementary Figure S5. Performance of the 17 differential expression methods based on the three FDR cutoffs proposed in this work. (**a**) FDR < 0.05, (**b)** FDR < 0.01 and (**c**) FDR < 0.001.

	CLA-T0 vs. CLB-T0			CLA-T1 vs. CLA-T0			CLA-T2 vs. CLA-T0			CLB-T1 vs. CLB-T0			CLB-T2 vs. CLB-T0			Overall median
	FDR < 0.05	FDR < 0.01	FDR < 0.001	FDR < 0.05	FDR < 0.01	FDR < 0.001	FDR < 0.05	FDR < 0.01	FDR < 0.001	FDR < 0.05	FDR < 0.01	FDR < 0.001	FDR < 0.05	FDR < 0.01	FDR < 0.001	
**Ballgown**	12	10	8	17	7	14	12	16	6	6	5	11	5	14	16	**11**
**baySeq**	12	9	7	14	14	2	12	6	3	3	1	1	7	4	5	**6**
**Cuffdiff**	9	11	9	10	5	1	8	13	5	17	14	5	17	5	13	**9**
**DESeq2**	1	1	11	3	11	11	17	13	11	13	12	14	9	13	6	**11**
**EBSeq**	11	6	13	11	6	9	15	15	17	7	2	4	1	1	1	**7**
**edgeR exact RLE**	8	1	11	3	11	11	16	12	7	15	9	14	12	12	14	**11**
**edgeR exact TMM**	1	14	6	6	13	15	10	8	12	13	9	14	12	10	15	**12**
**edgeR exact UQ**	3	13	10	1	8	17	2	11	14	16	11	14	3	10	10	**10**
**edgeR GLM RLE**	15	3	4	6	16	7	11	3	13	9	7	7	16	7	9	**7**
**edgeR GLM TMM**	4	3	5	5	2	6	9	10	9	9	7	12	14	7	11	**7**
**edgeR GLM UQ**	4	5	3	8	17	13	2	16	16	8	6	2	4	9	11	**8**
**limma trend**	7	7	1	11	9	4	6	7	15	11	3	3	14	3	6	**7**
**limma voom**	4	12	2	11	9	10	6	8	7	12	3	5	11	2	6	**7**
**NOISeq FPKM**	17	8	14	8	1	8	1	1	1	5	13	8	10	6	2	**8**
**NOISeq TMM**	10	15	15	2	3	5	12	4	9	2	16	10	2	15	4	**9**
**NOISeq UQ**	14	15	16	16	4	2	4	2	2	4	17	9	6	16	2	**6**
**SAMseq**	16	17	17	15	14	16	5	5	4	1	15	13	8	17	16	**15**

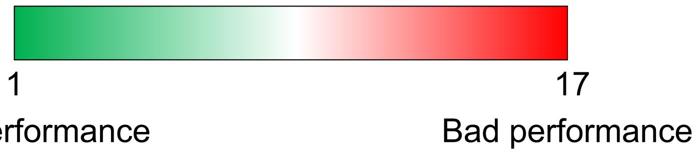

Supplementary Figure S6. Overall performance of the 17 differential expression methods based on the combination of the five contrasts and the three FDR cutoffs proposed in this work. CLA is the KMS12-BM cell line, CLB is the JJN-3 cell line, T0 is DMSO treatment, T1 is Amiloride treatment and T2 is TG003 treatment.

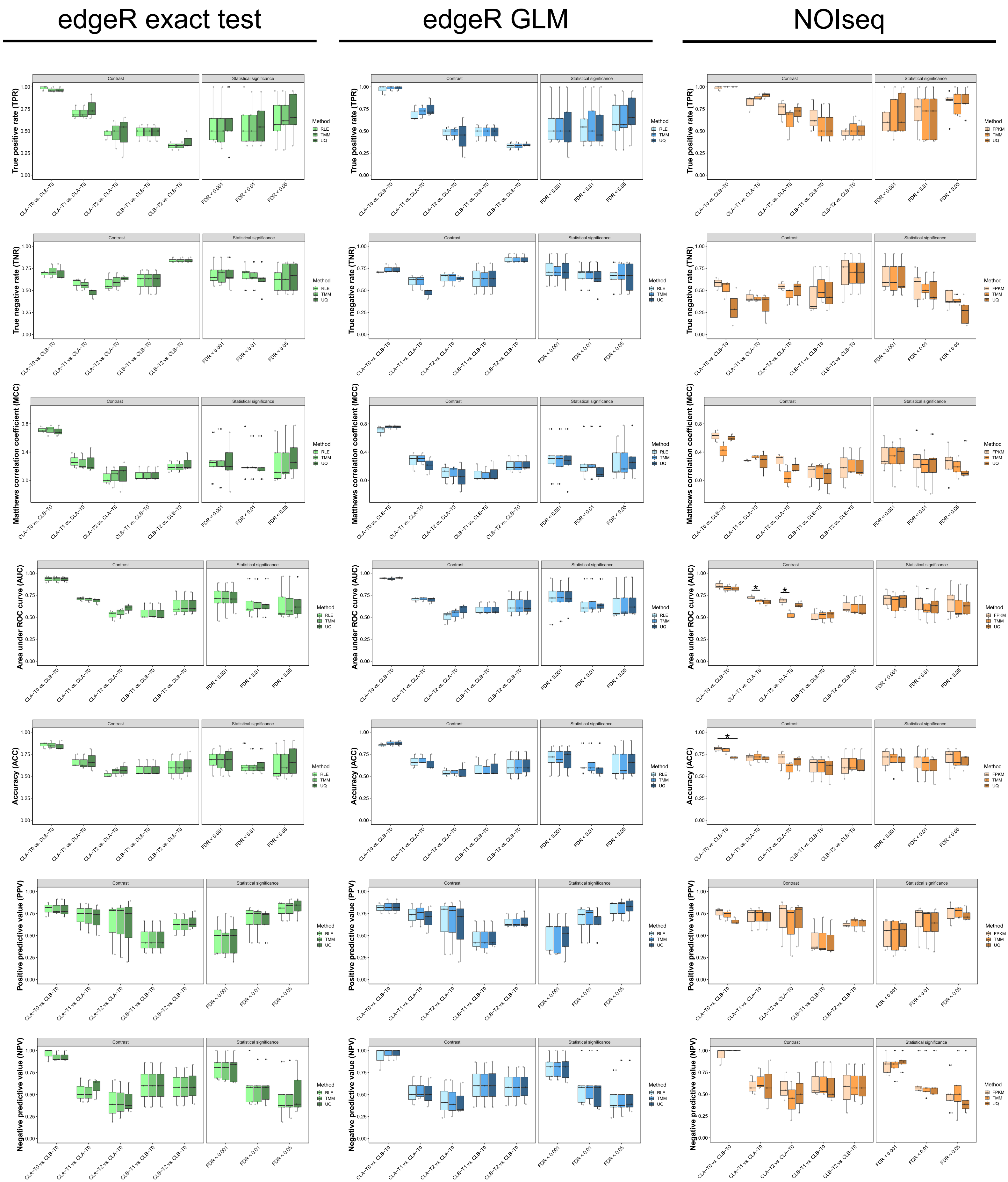

Supplementary Figure S7: Influence of normalization on differentially gene expression detection. Boxplot graphs were performed for the differential expression methods that admitted multiple normalization approaches (edgeR exact test, edgeR GLM and NOIseq) and represent the values of 7 diagnostic test parameters: true positive rate (TPR), true negative rate (TNR), Matthews correlation coefficient (MCC), accuracy (ACC), area under the ROC curve (AUC), positive predictive value (PPV) and negative predictive value. These parameters were measured at three FDR cut-off levels and five experimental contrasts, which are the result of the combination of two cell lines (CLA and CLB) with two treatment options (T1 and T2) and DMSO (T0).The asterisks indicate the significance of the post-hoc Dunn's test: * $p < 0.05$, ** $p < 0.01$ and *** $p < 0.001$. Comparisons without asterisk are statistically insignificant ($p > 0.05$).

Supplementary Table S1. Initial number of reads for each of the analysed samples.

Sample	Input paired reads	Reads Q > 20		
		R1	R2	Mean R1 R2
CLA-T0-S1	50,946,237	50,042,477	49,158,906	49,600,691.5
CLA-T0-S2	62,571,514	60,745,925	59,593,922	60,169,923.5
CLA-T0-S3	77,906,369	75,745,249	74,632,834	75,189,041.5
CLB-T0-S1	46,810,430	46,097,180	45,536,869	45,817,024.5
CLB-T0-S2	60,376,359	58,310,725	57,124,153	57,717,439
CLB-T0-S3	40,840,185	39,720,525	39,264,085	39,492,305
CLA-T1-S1	60,733,681	58,485,539	56,777,315	57,631,427
CLA-T1-S2	66,024,375	64,206,003	63,222,987	63,714,495
CLA-T1-S3	45,598,113	45,138,641	44,429,554	44,784,097.5
CLB-T1-S1	37,568,155	36,970,927	36,711,895	36,841,411
CLB-T1-S2	58,656,767	57,180,047	56,287,686	56,733,866.5
CLB-T1-S3	64,447,257	62,685,537	62,013,794	62,349,665.5
CLA-T2-S1	48,670,462	46,934,263	45,967,599	46,450,931
CLA-T2-S2	40,156,676	39,052,670	38,459,170	38,755,920
CLA-T2-S3	49,142,091	48,615,422	47,902,731	48,259,076.5
CLB-T2-S1	36,240,231	35,637,569	34,837,372	35,237,470.5
CLB-T2-S2	59,666,947	57,558,513	56,118,366	56,838,439.5
CLB-T2-S3	41,733,924	40,510,376	39,886,662	40,198,519

Supplementary Table S2. List of the 107 genes selected for the pipeline precision analysis and the 32 genes selected (highlighted genes) for the pipeline accuracy analysis.

Selected for qRT-PCR?	Ensembl ID	HGNC symbol	Chromosome	Gene type	Median MAD ranking
Selected	ENSG00000100138	NHP2L1	22	protein coding	7
Selected	ENSG00000105193	RPS16	19	protein coding	7
Selected	ENSG00000068697	LAPTM4A	2	protein coding	8
Selected	ENSG00000127884	ECHS1	10	protein coding	10
Selected	ENSG00000101361	NOP56	20	protein coding	14
Selected	ENSG00000165502	RPL36AL	14	protein coding	14.5
Selected	ENSG00000154723	ATP5J	21	protein coding	16
Selected	ENSG00000143158	MPC2	1	protein coding	16
Selected	ENSG00000138279	ANXA7	10	protein coding	17
Selected	ENSG00000107223	EDF1	9	protein coding	17
NO	ENSG00000163131	CTSS	1	protein coding	17.5
NO	ENSG00000156467	UQCRB	8	protein coding	18.5
NO	ENSG00000130255	RPL36	19	protein coding	19
NO	ENSG00000178913	TAF7	5	protein coding	20
NO	ENSG00000198961	PJA2	5	protein coding	20.5
NO	ENSG00000092820	EZR	6	protein coding	21
NO	ENSG00000114391	RPL24	3	protein coding	21
Selected	ENSG00000142541	RPL13A	19	protein coding	22
NO	ENSG00000164587	RPS14	5	protein coding	22
NO	ENSG00000156482	RPL30	8	protein coding	23
NO	ENSG00000151366	NDUFC2	11	protein coding	24
NO	ENSG00000164919	COX6C	8	protein coding	24.5
NO	ENSG00000172115	CYCS	7	protein coding	25.5
NO	ENSG00000100348	TXN2	22	protein coding	26
Selected	ENSG00000147677	EIF3H	8	protein coding	28
NO	ENSG00000183978	COA3	17	protein coding	29
NO	ENSG00000125743	SNRPD2	19	protein coding	29
NO	ENSG00000265681	RPL17	18	protein coding	30
NO	ENSG00000169976	SF3B5	6	protein coding	30
NO	ENSG00000198242	RPL23A	17	protein coding	31
NO	ENSG00000178952	TUFM	16	protein coding	33
NO	ENSG00000122566	HNRNPA2B1	7	protein coding	34
NO	ENSG00000108107	RPL28	19	protein coding	34
Selected	ENSG00000166710	B2M	15	protein coding	35
NO	ENSG00000170144	HNRNPA3	2	protein coding	35
NO	ENSG00000196776	CD47	3	protein coding	36
NO	ENSG00000099795	NDUFB7	19	protein coding	37
NO	ENSG00000177700	POLR2L	11	protein coding	37
NO	ENSG00000117614	SYF2	1	protein coding	38
NO	ENSG00000115053	NCL	2	protein coding	39
Selected	ENSG00000161016	RPL8	8	protein coding	39
NO	ENSG00000165119	HNRNPK	9	protein coding	40
NO	ENSG00000244038	DDOST	1	protein coding	42
NO	ENSG00000145592	RPL37	5	protein coding	42
NO	ENSG00000147604	RPL7	8	protein coding	43
NO	ENSG00000105583	WDR83OS	19	protein coding	43
NO	ENSG00000164032	H2AFZ	4	protein coding	44
NO	ENSG00000088832	FKBP1A	20	protein coding	45
NO	ENSG00000070756	PABPC1	8	protein coding	45
NO	ENSG00000115128	SF3B14	2	protein coding	46.5
Selected	ENSG00000131495	NDUFA2	5	protein coding	48
NO	ENSG00000096384	HSP90AB1	6	protein coding	50

NO	ENSG00000169714	CNBP	3	protein coding	51
NO	ENSG00000114850	SSR3	3	protein coding	54
NO	ENSG00000118418	HMGN3	6	protein coding	55
NO	ENSG00000153187	HNRNPU	1	protein coding	56
NO	ENSG00000143162	CREG1	1	protein coding	57
NO	ENSG00000144713	RPL32	3	protein coding	57
NO	ENSG00000125835	SNRPB	20	protein coding	57
NO	ENSG00000143106	PSMA5	1	protein coding	59
Selected	ENSG00000142937	RPS8	1	protein coding	59
NO	ENSG00000135486	HNRNPA1	12	protein coding	61
NO	ENSG00000132646	PCNA	20	protein coding	61
NO	ENSG00000174444	RPL4	15	protein coding	64
NO	ENSG00000154518	ATP5G3	2	protein coding	66
NO	ENSG00000181163	NPM1	5	protein coding	66
NO	ENSG00000188612	SUMO2	17	protein coding	66
Selected	ENSG00000166337	TAF10	11	protein coding	66
NO	ENSG00000170027	YWHAG	7	protein coding	67.5
NO	ENSG00000143612	C1orf43	1	protein coding	68
NO	ENSG00000239264	TXNDC5	6	protein coding	68
NO	ENSG00000164096	C4orf3	4	protein coding	72
NO	ENSG00000152795	HNRNPDL	4	protein coding	74
NO	ENSG00000134825	TMEM258	11	protein coding	74
NO	ENSG00000115484	CCT4	2	protein coding	75
NO	ENSG00000142864	SERBP1	1	protein coding	76
Selected	ENSG00000107581	EIF3A	10	protein coding	78
NO	ENSG00000116251	RPL22	1	protein coding	78
NO	ENSG00000103363	TCEB2	16	protein coding	78
NO	ENSG00000134884	ARGLU1	13	protein coding	79
NO	ENSG00000185043	CIB1	15	protein coding	81
NO	ENSG00000166441	RPL27A	11	protein coding	81
NO	ENSG00000069275	NUCKS1	1	protein coding	82
NO	ENSG00000170348	TMED10	14	protein coding	82
NO	ENSG00000108518	PFN1	17	protein coding	83
NO	ENSG00000111897	SERINC1	6	protein coding	83
Selected	ENSG00000008018	PSMB1	6	protein coding	86
NO	ENSG00000196262	PPIA	7	protein coding	87
NO	ENSG00000137154	RPS6	9	protein coding	88
NO	ENSG00000111639	MRPL51	12	protein coding	89
NO	ENSG00000173113	TRMT112	11	protein coding	89
NO	ENSG00000152518	ZFP36L2	2	protein coding	89
NO	ENSG00000106153	CHCHD2	7	protein coding	93
NO	ENSG00000124767	GLO1	6	protein coding	93
Selected	ENSG00000178741	COX5A	15	protein coding	94
NO	ENSG00000133639	BTG1	12	protein coding	95.5
NO	ENSG00000166562	SEC11C	18	protein coding	96
Selected	ENSG00000044574	HSPA5	9	protein coding	97
Selected	ENSG00000115758	ODC1	2	protein coding	98
Selected	ENSG00000182117	NOP10	15	protein coding	100
Selected	ENSG00000119421	NDUFA8	9	protein coding	101
Selected	ENSG00000168264	IRF2BP2	1	protein coding	102
Selected	ENSG00000163956	LRPAP1	4	protein coding	102
Selected	ENSG00000065978	YBX1	1	protein coding	104
Selected	ENSG00000105968	H2AFV	7	protein coding	105
Selected	ENSG00000105887	MTPN	7	protein coding	106
Selected	ENSG00000175130	MARCKSL1	1	protein coding	107
**Selected**	**ENSG0000007562**	**ACTB**	**7**	**Housekeeping gene**	

**Selected**	**ENSG00000111640**	**GAPDH**	**12**	**Housekeeping gene**	

*Supplementary Table S2 legend:

**Genes selected by the median absolute deviation (MAD) among all control samples:**	**Low MAD**
	**High MAD**
**Randomly selected genes**	
**Selected Housekeeping genes**	

Suplementary Table S3. Oligonucleotide probes used for the TaqMan qRT-PCR assay.

Ensembl ID	HGNC ID	Chromosome	TaqMan Gene Expression ID assay	Source	Identifier
ENSG00000008018	PSMB1	6	Hs00427357_m1	ThermoFisher	Cat#4448892
ENSG00000044574	HSPA5	9	Hs00607129_gH	ThermoFisher	Cat#4448892
ENSG00000065978	YBX1	1	Hs00358903_g1	ThermoFisher	Cat#4448892
ENSG00000068697	LAPTM4A	2	Hs01092025_m1	ThermoFisher	Cat#4448892
ENSG0000007562	ACTB	7	Hs01060665_g1	ThermoFisher	Cat#4448892
ENSG00000100138	NHP2L1 (SNU13)	22	Hs03025442_s1	ThermoFisher	Cat#4448892
ENSG00000101361	NOP56	20	Hs00197340_m1	ThermoFisher	Cat#4448892
ENSG00000105193	RPS16	19	Hs01598518_gH	ThermoFisher	Cat#4448892
ENSG00000105887	MTPN	7	Hs00377581_m1	ThermoFisher	Cat#4448892
ENSG00000105968	H2AFV	7	Hs00606542_mH	ThermoFisher	Cat#4448892
ENSG00000107223	EDF1	9	Hs00610152_m1	ThermoFisher	Cat#4448892
ENSG00000107581	EIF3A	10	Hs01025769_m1	ThermoFisher	Cat#4448892
ENSG00000111640	GAPDH	12	Hs02786624_g1	ThermoFisher	Cat#4453320
ENSG00000115758	ODC1	2	Hs00159739_m1	ThermoFisher	Cat#4453320
ENSG00000119421	NDUFA8	9	Hs00204417_m1	ThermoFisher	Cat#4448892
ENSG00000127884	ECHS1	10	Hs00187943_m1	ThermoFisher	Cat#4448892
ENSG00000131495	NDUFA2	5	Hs04187282_g1	ThermoFisher	Cat#4448892
ENSG00000138279	ANXA7	10	Hs00559410_m1	ThermoFisher	Cat#4448892
ENSG00000142541	RPL13A	19	Hs04194366_g1	ThermoFisher	Cat#4448892
ENSG00000142937	RPS8	1	Hs01374307_g1	ThermoFisher	Cat#4448892
ENSG00000143158	MPC2	1	Hs00967250_m1	ThermoFisher	Cat#4448892
ENSG00000147677	EIF3H	8	Hs00186779_m1	ThermoFisher	Cat#4448892
ENSG00000154723	ATP5J	21	Hs01081389_g1	ThermoFisher	Cat#4448892
ENSG00000161016	RPL8	8	Hs00361285_g1	ThermoFisher	Cat#4453320
ENSG00000163956	LRPAP1	4	Hs00936301_m1	ThermoFisher	Cat#4448892
ENSG00000165502	RPL36AL	14	Hs00733231_m1	ThermoFisher	Cat#4448892
ENSG00000166337	TAF10	11	Hs00359540_g1	ThermoFisher	Cat#4448892
ENSG00000166710	B2M	15	Hs00187842_m1	ThermoFisher	Cat#4453320
ENSG00000168264	IRF2BP2	1	Hs02930738_m1	ThermoFisher	Cat#4448892
ENSG00000175130	MARCKSL1	1	Hs00702769_s1	ThermoFisher	Cat#4448892
ENSG00000178741	COX5A	15	Hs00362067_m1	ThermoFisher	Cat#4448892
ENSG00000182117	NOP10	15	Hs00430282_m1	ThermoFisher	Cat#4448892

Supplementary Table S4. Precision analysis results

Median precision (cell lines A and B)						
**RANK position**	**Median RANK**	**MAD**	**Trimming method**	**Alignment method**	**Counting method**	**Normalization method**
**1**	48.5	0.5	Cutadapt	Salmon-QMB-AUT		TPM
**2**	49	0	Cutadapt	Salmon-FMD-AUT		TPM
**3**	49	1	Cutadapt	Salmon-FMD-ISR		TPM
**4**	50	1	Cutadapt	Salmon-QMB-ISR		TPM
**5**	50.5	0.5	Trimmomatic	Salmon-QMB-AUT		TPM
**6**	51.5	1.5	Cutadapt	Bowtie2	RSEM	TPM
**7**	51.5	4.5	BBDuk	Salmon-FMD-ISR		TPM
**8**	53.5	1.5	Trimmomatic	Salmon-QMB-ISR		TPM
**9**	53.5	4.5	Trimmomatic	Salmon-FMD-AUT		TPM
**10**	53.5	7.5	BBDuk	Salmon-FMD-AUT		TPM
**11**	54	3	Cutadapt	TopHat2	HTSeq INTER	TMM
**12**	54	4	BBDuk	Sailfish-ISR		TPM
**13**	54	6	Trimmomatic	STAR	RSEM	TPM
**14**	54.5	2.5	BBDuk	Salmon-QMB-AUT		TPM
**15**	55	3	Trimmomatic	Salmon-FMD-ISR		TPM
**16**	55	4	Trimmomatic	Bowtie2	RSEM	TPM
**17**	55	5	BBDuk	Salmon-QMB-ISR		TPM
**18**	56.5	2.5	Cutadapt	Sailfish-ISR		TPM
**19**	56.5	3.5	Cutadapt	Kallisto		TPM
**20**	56.5	4.5	BBDuk	Bowtie2	RSEM	TPM
**21**	57	5	Cutadapt	STAR	RSEM	TPM
**22**	58	10	BBDuk	Kallisto		TPM
**23**	58.5	7.5	BBDuk	TopHat2	HTSeq INTER	RLE
**24**	60.5	5.5	BBDuk	TopHat2	HTSeq UNION	RLE
**25**	61	1	BBDuk	STAR	Stringtie	FPKM
**26**	61	4	BBDuk	STAR	RSEM	TPM
**27**	61.5	0.5	Trimmomatic	STAR	Stringtie	FPKM
**28**	61.5	2.5	Trimmomatic	TopHat2	Cufflinks	FPKM
**29**	61.5	8.5	Trimmomatic	TopHat2	HTSeq INTER	RLE
**30**	62	2	Cutadapt	HiSat2	HTSeq UNION	TMM
**31**	62	4	Cutadapt	STAR	Stringtie	FPKM
**32**	62	4	Cutadapt	TopHat2	HTSeq UNION	RLE
**33**	62.5	7.5	Trimmomatic	TopHat2	HTSeq UNION	RLE
**34**	62.5	7.5	Trimmomatic	Kallisto		TPM
**35**	62.5	9.5	BBDuk	TopHat2	HTSeq INTER	TMM
**36**	62.5	12.5	Cutadapt	TopHat2	HTSeq UNION	TMM
**37**	63	0	Cutadapt	TopHat2	Cufflinks	FPKM
**38**	63	3	Cutadapt	HiSat2	HTSeq UNION	RLE
**39**	63	6	BBDuk	HiSat2	HTSeq INTER	TMM
**40**	63	8	Cutadapt	HiSat2	HTSeq INTER	RLE

**41**	63	11	Trimmomatic	HiSat2	HTSeq INTER	RLE
**42**	63.5	4.5	Trimmomatic	STAR	HTSeq INTER	TMM
**43**	63.5	5.5	Trimmomatic	RUM	HTSeq UNION	RLE
**44**	63.5	10.5	BBDuk	HiSat2	HTSeq UNION	TMM
**45**	63.5	12.5	BBDuk	TopHat2	HTSeq UNION	TMM
**46**	64	1	Trimmomatic	Bowtie2	RSEM	FPKM
**47**	64	2	BBDuk	STAR	Stringtie	TPM
**48**	64	3	BBDuk	STAR	Cufflinks	FPKM
**49**	64	9	Cutadapt	TopHat2	HTSeq INTER	RLE
**50**	64	10	BBDuk	HiSat2	HTSeq INTER	RLE
**51**	64.5	5.5	Trimmomatic	HiSat2	HTSeq INTER	TMM
**52**	64.5	7.5	BBDuk	STAR	HTSeq INTER	RLE
**53**	65	3	Cutadapt	Bowtie2	RSEM	FPKM
**54**	65	5	BBDuk	Bowtie2	RSEM	FPKM
**55**	65	6	BBDuk	HiSat2	HTSeq UNION	RLE
**56**	65	7	Trimmomatic	HiSat2	HTSeq UNION	RLE
**57**	65	11	Trimmomatic	Sailfish-ISR		TPM
**58**	65.5	0.5	Trimmomatic	STAR	RSEM	FPKM
**59**	65.5	1.5	BBDuk	HiSat2	Cufflinks	FPKM
**60**	65.5	6.5	Trimmomatic	HiSat2	HTSeq UNION	TMM
**61**	65.5	9.5	Trimmomatic	TopHat2	HTSeq INTER	TMM
**62**	66	1	Cutadapt	STAR	RSEM	FPKM
**63**	66	9	Trimmomatic	RUM	HTSeq INTER	RLE
**64**	66.5	2.5	BBDuk	TopHat2	Cufflinks	FPKM
**65**	67.5	0.5	BBDuk	STAR	RSEM	FPKM
**66**	67.5	4.5	Cutadapt	STAR	Stringtie	TPM
**67**	67.5	5.5	Cutadapt	STAR	HTSeq UNION	TMM
**68**	67.5	12.5	Trimmomatic	STAR	Stringtie	TPM
**69**	68	3	Trimmomatic	STAR	Cufflinks	FPKM
**70**	68	3	BBDuk	STAR	HTSeq UNION	RLE
**71**	68	7	BBDuk	STAR	HTSeq UNION	TMM
**72**	68.5	7.5	Trimmomatic	RUM	HTSeq INTER	TMM
**73**	68.5	8.5	Trimmomatic	RUM	HTSeq UNION	TMM
**74**	69	8	Trimmomatic	STAR	HTSeq UNION	TMM
**75**	69	9	Trimmomatic	TopHat2	HTSeq UNION	TMM
**76**	70	1	Trimmomatic	HiSat2	Cufflinks	FPKM
**77**	70	10	BBDuk	RUM	HTSeq UNION	TMM
**78**	70.5	9.5	Cutadapt	STAR	Cufflinks	FPKM
**79**	71	6	Trimmomatic	STAR	HTSeq INTER	RLE
**80**	71	8	BBDuk	STAR	HTSeq INTER	TMM
**81**	72	13	Cutadapt	RUM	Cufflinks	FPKM
**82**	72.5	9.5	Trimmomatic	RUM	Cufflinks	FPKM
**83**	73	5	BBDuk	RUM	HTSeq INTER	TMM
**84**	73.5	20.5	Cutadapt	STAR	HTSeq INTER	TMM
**85**	74	4	Cutadapt	HiSat2	Cufflinks	FPKM

**86**	74	11	Trimmomatic	STAR	HTSeq UNION	RLE
**87**	75	2	Cutadapt	HiSat2	HTSeq INTER	TMM
**88**	75.5	14.5	BBDuk	TopHat2	Stringtie	TPM
**89**	76	0	BBDuk	TopHat2	Stringtie	FPKM
**90**	76	1	Cutadapt	TopHat2	Stringtie	FPKM
**91**	76.5	6.5	Cutadapt	TopHat2	Stringtie	TPM
**92**	78.5	2.5	Trimmomatic	TopHat2	Stringtie	FPKM
**93**	78.5	8.5	Trimmomatic	TopHat2	Stringtie	TPM
**94**	80	2	BBDuk	RUM	HTSeq UNION	RLE
**95**	80.5	19.5	BBDuk	RUM	Cufflinks	FPKM
**96**	82	0	BBDuk	HiSat2	Stringtie	FPKM
**97**	82	0	BBDuk	RUM	HTSeq INTER	RLE
**98**	82.5	9.5	Cutadapt	STAR	HTSeq INTER	RLE
**99**	84.5	1.5	BBDuk	RUM	Stringtie	FPKM
**100**	84.5	3.5	BBDuk	HiSat2	Stringtie	TPM
**101**	84.5	9.5	Cutadapt	RUM	Stringtie	TPM
**102**	85	6	Cutadapt	RUM	HTSeq INTER	TMM
**103**	85	11	Cutadapt	STAR	HTSeq UNION	RLE
**104**	85.5	1.5	BBDuk	RUM	Stringtie	TPM
**105**	86	6	Cutadapt	RUM	HTSeq UNION	TMM
**106**	86.5	2.5	Trimmomatic	RUM	Stringtie	FPKM
**107**	87	4	Cutadapt	HiSat2	Stringtie	FPKM
**108**	87.5	2.5	Cutadapt	RUM	HTSeq INTER	RLE
**109**	87.5	4.5	Cutadapt	RUM	HTSeq UNION	RLE
**110**	87.5	4.5	Cutadapt	RUM	Stringtie	FPKM
**111**	88	5	Trimmomatic	HiSat2	Stringtie	FPKM
**112**	88.5	3.5	Cutadapt	HiSat2	Stringtie	TPM
**113**	90	4	Trimmomatic	HiSat2	Stringtie	TPM
**114**	90	4	Trimmomatic	RUM	Stringtie	TPM
**115**	102.5	8.5	Cutadapt	STAR	eXpress	TPM
**116**	103.5	6.5	Cutadapt	STAR	eXpress	FPKM
**117**	109	2	BBDuk	STAR	eXpress	FPKM
**118**	109.5	0.5	Trimmomatic	STAR	eXpress	TPM
**119**	112.5	2.5	Trimmomatic	STAR	eXpress	FPKM
**120**	113	3	BBDuk	STAR	eXpress	TPM
**121**	113.5	9.5	Cutadapt	Bowtie2	eXpress	FPKM
**122**	114	6	Cutadapt	Bowtie2	eXpress	TPM
**123**	118.5	3.5	BBDuk	Bowtie2	eXpress	TPM
**124**	118.5	4.5	BBDuk	Bowtie2	eXpress	FPKM
**125**	119	4	Trimmomatic	Bowtie2	eXpress	TPM
**126**	120.5	0.5	Trimmomatic	Bowtie2	eXpress	FPKM
**127**	139.5	11.5	BBDuk	RUM	Stringtie	Coverage
**128**	140	6	BBDuk	STAR	eXpress	Eff_Counts
**129**	140	10	Trimmomatic	HiSat2	HTSeq INTER	Raw
**130**	141	3	BBDuk	HiSat2	Stringtie	Coverage

**131**	141	11	Trimmomatic	HiSat2	HTSeq UNION	Raw
**132**	141	14	Trimmomatic	RUM	Stringtie	Coverage
**133**	141.5	2.5	BBDuk	TopHat2	Stringtie	Coverage
**134**	141.5	5.5	BBDuk	STAR	eXpress	Est_Count
**135**	142	10	BBDuk	Bowtie2	eXpress	Eff_Counts
**136**	143	6	BBDuk	HiSat2	HTSeq INTER	Raw
**137**	143	10	BBDuk	Salmon-FMD-ISR		NumReads
**138**	143	11	BBDuk	Salmon-FMD-AUT		NumReads
**139**	143.5	0.5	BBDuk	STAR	Stringtie	Coverage
**140**	143.5	7.5	BBDuk	HiSat2	HTSeq UNION	Raw
**141**	144.5	3.5	BBDuk	Bowtie2	eXpress	Est_Count
**142**	145	3	BBDuk	Kallisto		Est-Counts
**143**	145.5	18.5	Trimmomatic	Sailfish-ISR		NumReads
**144**	146	4	BBDuk	STAR	HTSeq INTER	Raw
**145**	146	5	BBDuk	Salmon-QMB-AUT		NumReads
**146**	146	9	Trimmomatic	STAR	eXpress	Eff_Counts
**147**	146	13	Trimmomatic	HiSat2	Stringtie	Coverage
**148**	146.5	3.5	BBDuk	Sailfish-ISR		NumReads
**149**	146.5	4.5	BBDuk	TopHat2	HTSeq INTER	Raw
**150**	147	4	BBDuk	Salmon-QMB-ISR		NumReads
**151**	147	7	BBDuk	TopHat2	HTSeq UNION	Raw
**152**	147.5	5.5	BBDuk	STAR	HTSeq UNION	Raw
**153**	147.5	13.5	Trimmomatic	RUM	HTSeq INTER	Raw
**154**	148.5	16.5	Trimmomatic	TopHat2	Stringtie	Coverage
**155**	149	6	Trimmomatic	STAR	eXpress	Est_Count
**156**	149	13	Trimmomatic	RUM	HTSeq UNION	Raw
**157**	151	9	Trimmomatic	Salmon-FMD-ISR		NumReads
**158**	151.5	8.5	Trimmomatic	Salmon-FMD-AUT		NumReads
**159**	151.5	8.5	BBDuk	RUM	HTSeq INTER	Raw
**160**	154	11	Trimmomatic	Kallisto		Est-Counts
**161**	154.5	7.5	BBDuk	RUM	HTSeq UNION	Raw
**162**	154.5	9.5	Trimmomatic	Salmon-QMB-ISR		NumReads
**163**	155	11	Trimmomatic	TopHat2	HTSeq INTER	Raw
**164**	155.5	12.5	Trimmomatic	STAR	HTSeq INTER	Raw
**165**	156	13	Trimmomatic	STAR	Stringtie	Coverage
**166**	156.5	2.5	Cutadapt	STAR	eXpress	Est_Count
**167**	157	9	Trimmomatic	TopHat2	HTSeq UNION	Raw
**168**	157	12	Trimmomatic	STAR	HTSeq UNION	Raw
**169**	157.5	0.5	Trimmomatic	Bowtie2	eXpress	Eff_Counts
**170**	159.5	0.5	Cutadapt	Kallisto		Est-Counts
**171**	160	8	Cutadapt	Salmon-FMD-AUT		NumReads
**172**	160.5	7.5	Cutadapt	Salmon-FMD-ISR		NumReads
**173**	162	3	Cutadapt	Sailfish-ISR		NumReads
**174**	162.5	4.5	Cutadapt	Salmon-QMB-AUT		NumReads

**175**	162.5	4.5	Cutadapt	Salmon-QMB-ISR		NumReads
**176**	162.5	6.5	Cutadapt	HiSat2	HTSeq INTER	Raw
**177**	163	7	Cutadapt	HiSat2	HTSeq UNION	Raw
**178**	163.5	5.5	Trimmomatic	Bowtie2	eXpress	Est_Count
**179**	164	7	Cutadapt	STAR	eXpress	Eff_Counts
**180**	164.5	0.5	Cutadapt	Bowtie2	eXpress	Est_Count
**181**	165	5	Cutadapt	RUM	Stringtie	Coverage
**182**	167.5	0.5	Cutadapt	HiSat2	Stringtie	Coverage
**183**	167.5	2.5	Cutadapt	TopHat2	Stringtie	Coverage
**184**	169.5	0.5	Cutadapt	RUM	HTSeq INTER	Raw
**185**	169.5	1.5	Cutadapt	STAR	Stringtie	Coverage
**186**	169.5	3.5	Cutadapt	Bowtie2	eXpress	Eff_Counts
**187**	170.5	4.5	Cutadapt	TopHat2	HTSeq INTER	Raw
**188**	171	1	Cutadapt	RUM	HTSeq UNION	Raw
**189**	172.5	0.5	Cutadapt	STAR	HTSeq INTER	Raw
**190**	173	1	Cutadapt	STAR	HTSeq UNION	Raw
**191**	173.5	4.5	Cutadapt	TopHat2	HTSeq UNION	Raw
**192**	177	12	Trimmomatic	Salmon-QMB-AUT		NumReads

Supplementary Table S5. Accuracy analysis results.

Median accuracy (cell lines A and B)						
**RANK position**	**Median RANK**	**MAD**	**Trimming method**	**Alignment method**	**Counting method**	**Normalization method**
**1**	27.5	18.5	Trimmomatic	Sailfish-ISR		NumReads
**2**	56	18.5	Trimmomatic	RUM	HTSeq UNION	TMM
**3**	56.5	30	BBDuk	RUM	HTSeq UNION	TMM
**4**	56.75	25.75	Cutadapt	RUM	HTSeq INTER	TMM
**5**	57.75	20.75	Trimmomatic	RUM	HTSeq INTER	TMM
**6**	58.25	24.25	Cutadapt	RUM	HTSeq UNION	TMM
**7**	62	32	BBDuk	RUM	HTSeq INTER	TMM
**8**	62	37.5	BBDuk	STAR	HTSeq UNION	TMM
**9**	64.75	8.25	Trimmomatic	STAR	HTSeq UNION	TMM
**10**	66.75	14.75	Trimmomatic	TopHat2	HTSeq UNION	TMM
**11**	67.75	22.75	Cutadapt	TopHat2	HTSeq UNION	TMM
**12**	68	23.5	BBDuk	TopHat2	HTSeq UNION	TMM
**13**	69.25	27.25	BBDuk	HiSat2	HTSeq UNION	TMM
**14**	69.5	20.5	Trimmomatic	HiSat2	HTSeq UNION	TMM
**15**	70.5	20.5	BBDuk	TopHat2	HTSeq INTER	TMM
**16**	71	12	Trimmomatic	STAR	HTSeq INTER	TMM
**17**	72.25	39.25	BBDuk	STAR	HTSeq INTER	TMM
**18**	72.5	24.5	Cutadapt	HiSat2	HTSeq INTER	TMM
**19**	73	19.5	Trimmomatic	TopHat2	HTSeq INTER	TMM
**20**	73.75	41.25	Trimmomatic	RUM	HTSeq UNION	RLE
**21**	76.25	26.75	Trimmomatic	HiSat2	HTSeq INTER	TMM
**22**	76.5	28	Cutadapt	STAR	HTSeq UNION	RLE
**23**	77.25	27.25	Cutadapt	STAR	HTSeq INTER	RLE
**24**	79	51	BBDuk	RUM	HTSeq UNION	RLE
**25**	80.25	51.75	BBDuk	RUM	HTSeq INTER	RLE
**26**	80.75	39.25	Trimmomatic	RUM	HTSeq INTER	RLE
**27**	81.75	36.75	Trimmomatic	RUM	Stringtie	Coverage
**28**	82.5	15.5	BBDuk	STAR	Stringtie	TPM
**29**	82.5	36	Cutadapt	RUM	Cufflinks	FPKM
**30**	82.75	13.25	Cutadapt	STAR	Cufflinks	FPKM
**31**	83.25	20.75	Cutadapt	STAR	HTSeq UNION	TMM
**32**	85.75	27.25	Trimmomatic	Bowtie2	eXpress	Eff_Counts
**33**	86	38.5	Trimmomatic	HiSat2	HTSeq INTER	Raw
**34**	86	43.5	Trimmomatic	RUM	HTSeq INTER	Raw
**35**	86.25	1.75	Cutadapt	STAR	HTSeq INTER	TMM
**36**	86.75	38.25	Trimmomatic	HiSat2	HTSeq UNION	Raw
**37**	87.25	44.75	Trimmomatic	RUM	HTSeq UNION	Raw
**38**	87.5	26.5	BBDuk	HiSat2	HTSeq INTER	TMM
**39**	88	41	Cutadapt	RUM	HTSeq INTER	RLE
**40**	88.5	32	BBDuk	RUM	Stringtie	Coverage

**41**	88.5	41.5	Trimmomatic	TopHat2	Stringtie	Coverage
**42**	88.75	19.25	Cutadapt	HiSat2	Cufflinks	FPKM
**43**	88.75	44.25	Cutadapt	RUM	HTSeq UNION	RLE
**44**	89.5	16	Trimmomatic	STAR	Cufflinks	FPKM
**45**	89.5	46.5	BBDuk	RUM	HTSeq INTER	Raw
**46**	89.75	40.75	Trimmomatic	HiSat2	Stringtie	Coverage
**47**	90	65	BBDuk	RUM	Cufflinks	FPKM
**48**	90.25	35.75	Trimmomatic	STAR	Stringtie	FPKM
**49**	90.25	61.25	BBDuk	STAR	HTSeq UNION	RLE
**50**	91	20	Trimmomatic	STAR	Stringtie	TPM
**51**	91.75	46.25	BBDuk	RUM	HTSeq UNION	Raw
**52**	92	19.5	Trimmomatic	HiSat2	Cufflinks	FPKM
**53**	92	42	BBDuk	STAR	Stringtie	FPKM
**54**	92	47.5	Trimmomatic	RUM	Cufflinks	FPKM
**55**	92.25	5.25	Trimmomatic	STAR	eXpress	FPKM
**56**	92.5	39.5	Trimmomatic	Kallisto		Est-Counts
**57**	93	30.5	Cutadapt	HiSat2	HTSeq UNION	TMM
**58**	93.5	44	Trimmomatic	Salmon-FMD-ISR		NumReads
**59**	93.75	24.25	Trimmomatic	STAR	HTSeq INTER	RLE
**60**	94	25	BBDuk	Bowtie2	eXpress	Eff_Counts
**61**	94.25	25.25	Trimmomatic	STAR	HTSeq UNION	RLE
**62**	94.25	43.75	Trimmomatic	Salmon-FMD-AUT		NumReads
**63**	94.25	47.25	Trimmomatic	Salmon-QMB-ISR		NumReads
**64**	94.25	49.75	BBDuk	HiSat2	HTSeq UNION	RLE
**65**	94.5	32	Trimmomatic	STAR	eXpress	Eff_Counts
**66**	94.75	28.25	Trimmomatic	Bowtie2	eXpress	Est_Count
**67**	94.75	38.75	Cutadapt	STAR	Stringtie	FPKM
**68**	94.75	41.25	Trimmomatic	STAR	Stringtie	Coverage
**69**	95	45	Trimmomatic	STAR	HTSeq INTER	Raw
**70**	95	46	Trimmomatic	TopHat2	HTSeq UNION	Raw
**71**	95	46.5	Trimmomatic	STAR	HTSeq UNION	Raw
**72**	95	61	BBDuk	STAR	HTSeq INTER	RLE
**73**	95.25	4.25	Cutadapt	HiSat2	Stringtie	TPM
**74**	95.5	33	Trimmomatic	STAR	eXpress	Est_Count
**75**	95.75	25.25	Cutadapt	STAR	Stringtie	TPM
**76**	96	45.5	Trimmomatic	TopHat2	HTSeq INTER	Raw
**77**	96.5	5.5	Cutadapt	RUM	Stringtie	TPM
**78**	97	21.5	BBDuk	Bowtie2	RSEM	FPKM
**79**	97.25	36.75	Cutadapt	HiSat2	HTSeq INTER	RLE
**80**	97.5	0	Trimmomatic	Bowtie2	eXpress	FPKM
**81**	97.5	52	BBDuk	HiSat2	HTSeq INTER	RLE
**82**	98	25	BBDuk	Bowtie2	eXpress	Est_Count
**83**	98.25	27.75	Cutadapt	STAR	RSEM	FPKM
**84**	98.75	28.75	BBDuk	HiSat2	Cufflinks	FPKM
**85**	99.25	20.75	Trimmomatic	HiSat2	Stringtie	FPKM

**86**	99.25	32.25	Trimmomatic	STAR	RSEM	FPKM
**87**	99.75	22.75	Cutadapt	Bowtie2	RSEM	FPKM
**88**	99.75	38.25	Cutadapt	HiSat2	HTSeq UNION	RLE
**89**	100	29.5	Trimmomatic	TopHat2	Cufflinks	FPKM
**90**	100.25	13.75	BBDuk	RUM	Stringtie	TPM
**91**	100.25	24.75	Trimmomatic	Bowtie2	RSEM	FPKM
**92**	101.25	24.75	BBDuk	STAR	Cufflinks	FPKM
**93**	101.25	31.25	BBDuk	TopHat2	Cufflinks	FPKM
**94**	101.5	26	BBDuk	STAR	RSEM	FPKM
**95**	101.5	46	BBDuk	TopHat2	HTSeq INTER	RLE
**96**	101.75	38.75	Cutadapt	RUM	Stringtie	FPKM
**97**	102	31	Cutadapt	TopHat2	Cufflinks	FPKM
**98**	102.5	37.5	BBDuk	TopHat2	Stringtie	Coverage
**99**	102.5	41	Trimmomatic	HiSat2	HTSeq UNION	RLE
**100**	102.75	4.25	BBDuk	HiSat2	Stringtie	TPM
**101**	102.75	10.75	Trimmomatic	HiSat2	Stringtie	TPM
**102**	102.75	35.25	BBDuk	RUM	Stringtie	FPKM
**103**	102.75	44.75	Trimmomatic	TopHat2	HTSeq INTER	RLE
**104**	102.75	49.25	Cutadapt	TopHat2	HTSeq INTER	RLE
**105**	103	24	Cutadapt	HiSat2	Stringtie	FPKM
**106**	103.25	0.75	Trimmomatic	Bowtie2	RSEM	TPM
**107**	103.25	2.75	Trimmomatic	TopHat2	Stringtie	TPM
**108**	103.25	36.25	BBDuk	HiSat2	Stringtie	Coverage
**109**	103.75	3.25	BBDuk	STAR	eXpress	FPKM
**110**	104	30	Trimmomatic	TopHat2	Stringtie	FPKM
**111**	104.25	28.75	BBDuk	HiSat2	Stringtie	FPKM
**112**	104.25	43.75	Trimmomatic	HiSat2	HTSeq INTER	RLE
**113**	104.5	1.5	Cutadapt	STAR	eXpress	FPKM
**114**	104.5	4	Cutadapt	Bowtie2	RSEM	TPM
**115**	104.5	8.5	Trimmomatic	RUM	Stringtie	TPM
**116**	105.25	6.75	BBDuk	Salmon-QMB-AUT		TPM
**117**	105.25	33.75	BBDuk	STAR	Stringtie	Coverage
**118**	105.25	44.75	BBDuk	HiSat2	HTSeq INTER	Raw
**119**	105.25	56.75	Cutadapt	RUM	Stringtie	Coverage
**120**	105.5	15.5	BBDuk	TopHat2	Stringtie	TPM
**121**	106	7	BBDuk	Salmon-QMB-ISR		TPM
**122**	106	10.5	BBDuk	Sailfish-ISR		TPM
**123**	106	13	Cutadapt	STAR	RSEM	TPM
**124**	106	47	BBDuk	TopHat2	HTSeq UNION	RLE
**125**	106	51	Cutadapt	TopHat2	HTSeq UNION	RLE
**126**	106.25	3.75	Trimmomatic	STAR	eXpress	TPM
**127**	106.25	16.25	BBDuk	Bowtie2	eXpress	FPKM
**128**	106.25	43.25	BBDuk	STAR	HTSeq INTER	Raw
**129**	106.5	61.5	Cutadapt	HiSat2	HTSeq INTER	Raw
**130**	106.75	44.25	Trimmomatic	TopHat2	HTSeq UNION	RLE

**131**	106.75	61.25	Cutadapt	HiSat2	HTSeq UNION	Raw
**132**	107	0	Cutadapt	TopHat2	Stringtie	TPM
**133**	107.75	21.75	Cutadapt	TopHat2	HTSeq INTER	TMM
**134**	107.75	27.75	BBDuk	STAR	eXpress	Eff_Counts
**135**	107.75	42.75	BBDuk	STAR	HTSeq UNION	Raw
**136**	108.25	31.25	Trimmomatic	RUM	Stringtie	FPKM
**137**	108.25	43.25	BBDuk	HiSat2	HTSeq UNION	Raw
**138**	108.5	6	BBDuk	Bowtie2	RSEM	TPM
**139**	108.5	11	Trimmomatic	Salmon-FMD-ISR		TPM
**140**	108.75	63.25	Cutadapt	HiSat2	Stringtie	Coverage
**141**	109	6.5	BBDuk	Salmon-FMD-AUT		TPM
**142**	109	7.5	Cutadapt	STAR	eXpress	TPM
**143**	109.25	31.75	Cutadapt	TopHat2	Stringtie	FPKM
**144**	109.25	61.75	Cutadapt	RUM	HTSeq UNION	Raw
**145**	109.5	7	BBDuk	Salmon-FMD-ISR		TPM
**146**	109.75	21.75	BBDuk	TopHat2	Stringtie	FPKM
**147**	110	11.5	Trimmomatic	Salmon-QMB-AUT		TPM
**148**	110	47.5	BBDuk	TopHat2	HTSeq INTER	Raw
**149**	110	49.5	BBDuk	Salmon-QMB-ISR		NumReads
**150**	110.25	49.25	BBDuk	Sailfish-ISR		NumReads
**151**	110.5	6.5	Trimmomatic	Bowtie2	eXpress	TPM
**152**	110.5	46.5	BBDuk	TopHat2	HTSeq UNION	Raw
**153**	110.5	48.5	BBDuk	Salmon-QMB-AUT		NumReads
**154**	110.5	52.5	Cutadapt	STAR	eXpress	Est_Count
**155**	110.75	19.25	BBDuk	STAR	RSEM	TPM
**156**	110.75	59.25	Cutadapt	RUM	HTSeq INTER	Raw
**157**	111	7.5	BBDuk	STAR	eXpress	TPM
**158**	111	10.5	Trimmomatic	Salmon-FMD-AUT		TPM
**159**	111.5	9	Trimmomatic	Salmon-QMB-ISR		TPM
**160**	112.25	54.25	Cutadapt	Bowtie2	eXpress	Est_Count
**161**	112.5	6	BBDuk	Bowtie2	eXpress	TPM
**162**	112.75	45.25	BBDuk	Kallisto		Est-Counts
**163**	113	14	Cutadapt	Salmon-QMB-ISR		TPM
**164**	113.5	57.5	Cutadapt	TopHat2	Stringtie	Coverage
**165**	113.5	63.5	Cutadapt	STAR	HTSeq UNION	Raw
**166**	113.75	61.75	Cutadapt	STAR	Stringtie	Coverage
**167**	114	25.5	BBDuk	STAR	eXpress	Est_Count
**168**	114	52.5	Cutadapt	STAR	eXpress	Eff_Counts
**169**	114.25	42.25	BBDuk	Salmon-FMD-ISR		NumReads
**170**	114.25	62.75	Cutadapt	STAR	HTSeq INTER	Raw
**171**	114.5	42.5	BBDuk	Salmon-FMD-AUT		NumReads
**172**	114.5	67	Cutadapt	Salmon-QMB-ISR		NumReads
**173**	114.75	10.75	Trimmomatic	STAR	RSEM	TPM
**174**	114.75	55.25	Cutadapt	Bowtie2	eXpress	Eff_Counts

**175**	115.25	14.25	Cutadapt	Salmon-QMB-AUT		TPM
**176**	115.25	66.25	Cutadapt	Salmon-QMB-AUT		NumReads
**177**	115.5	61	Cutadapt	TopHat2	HTSeq INTER	Raw
**178**	115.5	61	Cutadapt	TopHat2	HTSeq UNION	Raw
**179**	115.5	66	Cutadapt	Sailfish-ISR		NumReads
**180**	116.75	16.25	Cutadapt	Sailfish-ISR		TPM
**181**	117	9.5	Cutadapt	Salmon-FMD-ISR		TPM
**182**	117.5	16	Cutadapt	Bowtie2	eXpress	FPKM
**183**	117.75	63.25	Cutadapt	Salmon-FMD-ISR		NumReads
**184**	118	11	Cutadapt	Salmon-FMD-AUT		TPM
**185**	118.5	62.5	Cutadapt	Salmon-FMD-AUT		NumReads
**186**	118.75	59.75	Cutadapt	Kallisto		Est-Counts
**187**	119.25	30.25	BBDuk	Kallisto		TPM
**188**	119.75	72.25	Trimmomatic	Salmon-QMB-AUT		NumReads
**189**	120.25	25.25	Trimmomatic	Kallisto		TPM
**190**	125	25.5	Cutadapt	Kallisto		TPM
**191**	129.5	11	Trimmomatic	Sailfish-ISR		TPM
**192**	133.25	21.75	Cutadapt	Bowtie2	eXpress	TPM

Supplementary Table S6. Overall ranking of the 192 pipelines based on the sum of the precision and accuracy in the two cell lines.

Overall RANK (precision and accuracy, cell lines A and B)						
**RANK position**	**Sum RANK**	**MAD**	**Trimming method**	**Alignment method**	**Counting method**	**Normalization method**
**1**	249	8.5	Trimmomatic	RUM	HTSeq UNION	TMM
**2**	252.5	8.75	Trimmomatic	RUM	HTSeq INTER	TMM
**3**	253	13.25	BBDuk	RUM	HTSeq UNION	TMM
**4**	260	19.25	BBDuk	STAR	HTSeq UNION	TMM
**5**	260.5	15	Cutadapt	TopHat2	HTSeq UNION	TMM
**6**	263	15.75	BBDuk	TopHat2	HTSeq UNION	TMM
**7**	265.5	16	BBDuk	HiSat2	HTSeq UNION	TMM
**8**	266	11	BBDuk	TopHat2	HTSeq INTER	TMM
**9**	267.5	8	Trimmomatic	STAR	HTSeq UNION	TMM
**10**	269	4.5	Trimmomatic	STAR	HTSeq INTER	TMM
**11**	270	11.5	Trimmomatic	HiSat2	HTSeq UNION	TMM
**12**	270	13	BBDuk	RUM	HTSeq INTER	TMM
**13**	271.5	10.75	Trimmomatic	TopHat2	HTSeq UNION	TMM
**14**	274.5	18.25	Trimmomatic	RUM	HTSeq UNION	RLE
**15**	277	10.75	Trimmomatic	TopHat2	HTSeq INTER	TMM
**16**	281.5	10.25	Trimmomatic	HiSat2	HTSeq INTER	TMM
**17**	283.5	6	Cutadapt	RUM	HTSeq INTER	TMM
**18**	286.5	23	BBDuk	STAR	HTSeq INTER	TMM
**19**	288.5	6	Cutadapt	RUM	HTSeq UNION	TMM
**20**	293	2.5	BBDuk	STAR	Stringtie	TPM
**21**	293.5	16.75	Trimmomatic	RUM	HTSeq INTER	RLE
**22**	295	12	Cutadapt	HiSat2	HTSeq INTER	TMM
**23**	301	6	BBDuk	HiSat2	HTSeq INTER	TMM
**24**	301.5	5.5	Cutadapt	STAR	HTSeq UNION	TMM
**25**	303.5	3.75	Trimmomatic	STAR	Stringtie	FPKM
**26**	306	6	BBDuk	STAR	Stringtie	FPKM
**27**	306.5	9.5	Cutadapt	STAR	Cufflinks	FPKM
**28**	309	19.25	Cutadapt	RUM	Cufflinks	FPKM
**29**	310	2	Cutadapt	HiSat2	HTSeq UNION	TMM
**30**	312	25.25	Cutadapt	Bowtie2	RSEM	TPM
**31**	313.5	5	Cutadapt	STAR	Stringtie	FPKM
**32**	315	4.25	Trimmomatic	STAR	Cufflinks	FPKM
**33**	316.5	21	BBDuk	STAR	HTSeq UNION	RLE
**34**	316.5	22.5	Trimmomatic	Bowtie2	RSEM	TPM
**35**	317	12.5	Trimmomatic	STAR	Stringtie	TPM
**36**	318	26	BBDuk	RUM	HTSeq UNION	RLE
**37**	318.5	13.25	BBDuk	HiSat2	HTSeq UNION	RLE
**38**	319	19	BBDuk	STAR	HTSeq INTER	RLE
**39**	319.5	4.75	Cutadapt	STAR	HTSeq INTER	TMM

**40**	319.5	15.75	Cutadapt	STAR	HTSeq INTER	RLE
**41**	319.5	23.25	BBDuk	Salmon-QMB-AUT		TPM
**42**	320	7.5	BBDuk	TopHat2	HTSeq INTER	RLE
**43**	320	22.75	BBDuk	Sailfish-ISR		TPM
**44**	320.5	8	Cutadapt	HiSat2	HTSeq INTER	RLE
**45**	321	24.25	Trimmomatic	Salmon-QMB-AUT		TPM
**46**	322	24.5	BBDuk	Salmon-QMB-ISR		TPM
**47**	322	27.75	BBDuk	Salmon-FMD-ISR		TPM
**48**	323	5.75	Trimmomatic	TopHat2	Cufflinks	FPKM
**49**	323	14.25	BBDuk	HiSat2	HTSeq INTER	RLE
**50**	323	15.25	Cutadapt	STAR	HTSeq UNION	RLE
**51**	323.5	17.5	Cutadapt	TopHat2	HTSeq INTER	TMM
**52**	324	1.75	Trimmomatic	HiSat2	Cufflinks	FPKM
**53**	324	7.75	BBDuk	Bowtie2	RSEM	FPKM
**54**	324.5	25	BBDuk	RUM	HTSeq INTER	RLE
**55**	325	27.25	BBDuk	Salmon-FMD-AUT		TPM
**56**	325.5	3	Cutadapt	HiSat2	HTSeq UNION	RLE
**57**	325.5	4.25	Cutadapt	HiSat2	Cufflinks	FPKM
**58**	326	20.5	Cutadapt	STAR	RSEM	TPM
**59**	326	25	Cutadapt	Salmon-QMB-ISR		TPM
**60**	326.5	4.5	Cutadapt	STAR	Stringtie	TPM
**61**	327	22.75	Trimmomatic	Salmon-FMD-ISR		TPM
**62**	327.5	26.5	Cutadapt	Salmon-QMB-AUT		TPM
**63**	328.5	2.75	Cutadapt	STAR	RSEM	FPKM
**64**	328.5	3	BBDuk	HiSat2	Cufflinks	FPKM
**65**	328.5	6.25	Trimmomatic	Bowtie2	RSEM	FPKM
**66**	328.5	8.5	Trimmomatic	TopHat2	HTSeq INTER	RLE
**67**	329	18.75	Trimmomatic	RUM	Cufflinks	FPKM
**68**	329	25.75	Trimmomatic	Salmon-FMD-AUT		TPM
**69**	329.5	1	Trimmomatic	STAR	RSEM	FPKM
**70**	329.5	6	Trimmomatic	STAR	HTSeq INTER	RLE
**71**	329.5	7.5	Cutadapt	Bowtie2	RSEM	FPKM
**72**	330	4	Cutadapt	TopHat2	Cufflinks	FPKM
**73**	330	25.25	Trimmomatic	Salmon-QMB-ISR		TPM
**74**	330	25.25	BBDuk	Bowtie2	RSEM	TPM
**75**	330.5	7.75	BBDuk	STAR	Cufflinks	FPKM
**76**	332	29.75	Cutadapt	Salmon-FMD-ISR		TPM
**77**	333	5.5	BBDuk	TopHat2	HTSeq UNION	RLE
**78**	333.5	9.75	Cutadapt	TopHat2	HTSeq INTER	RLE
**79**	334	29	Cutadapt	Salmon-FMD-AUT		TPM
**80**	334.5	11	Trimmomatic	HiSat2	HTSeq INTER	RLE
**81**	335	7	Trimmomatic	HiSat2	HTSeq UNION	RLE
**82**	335.5	3	BBDuk	TopHat2	Cufflinks	FPKM
**83**	336	5.5	Cutadapt	TopHat2	HTSeq UNION	RLE
**84**	336.5	11	Trimmomatic	STAR	HTSeq UNION	RLE

**85**	337.5	28	Trimmomatic	STAR	RSEM	TPM
**86**	338	4.25	BBDuk	STAR	RSEM	FPKM
**87**	338.5	7.5	Trimmomatic	TopHat2	HTSeq UNION	RLE
**88**	341	37.5	BBDuk	RUM	Cufflinks	FPKM
**89**	343.5	17.25	BBDuk	STAR	RSEM	TPM
**90**	346	59	Trimmomatic	Sailfish-ISR		NumReads
**91**	346.5	23.25	Cutadapt	Sailfish-ISR		TPM
**92**	351	21.5	Cutadapt	RUM	HTSeq INTER	RLE
**93**	352.5	23.75	Cutadapt	RUM	HTSeq UNION	RLE
**94**	354.5	20.5	BBDuk	Kallisto		TPM
**95**	362	5.5	Cutadapt	RUM	Stringtie	TPM
**96**	362	14.5	BBDuk	TopHat2	Stringtie	TPM
**97**	363	23.25	Cutadapt	Kallisto		TPM
**98**	363.5	9.5	Trimmomatic	TopHat2	Stringtie	TPM
**99**	365	3.5	Trimmomatic	TopHat2	Stringtie	FPKM
**100**	365.5	20	Trimmomatic	Kallisto		TPM
**101**	367	12	Cutadapt	TopHat2	Stringtie	TPM
**102**	367.5	3.5	Cutadapt	HiSat2	Stringtie	TPM
**103**	370.5	1.25	Cutadapt	TopHat2	Stringtie	FPKM
**104**	371.5	1.5	BBDuk	RUM	Stringtie	TPM
**105**	371.5	6	BBDuk	TopHat2	Stringtie	FPKM
**106**	372.5	3.25	BBDuk	HiSat2	Stringtie	FPKM
**107**	374.5	7.25	Trimmomatic	HiSat2	Stringtie	FPKM
**108**	374.5	8.75	BBDuk	HiSat2	Stringtie	TPM
**109**	374.5	9.25	BBDuk	RUM	Stringtie	FPKM
**110**	378.5	14.5	Cutadapt	RUM	Stringtie	FPKM
**111**	380	6	Cutadapt	HiSat2	Stringtie	FPKM
**112**	385.5	4	Trimmomatic	HiSat2	Stringtie	TPM
**113**	389	5	Trimmomatic	RUM	Stringtie	TPM
**114**	389	32.25	Trimmomatic	Sailfish-ISR		TPM
**115**	389.5	6	Trimmomatic	RUM	Stringtie	FPKM
**116**	409.5	8.75	Trimmomatic	STAR	eXpress	FPKM
**117**	416	3.5	Cutadapt	STAR	eXpress	FPKM
**118**	423	7.5	Cutadapt	STAR	eXpress	TPM
**119**	425.5	2	BBDuk	STAR	eXpress	FPKM
**120**	431.5	0.5	Trimmomatic	STAR	eXpress	TPM
**121**	436	11.25	Trimmomatic	Bowtie2	eXpress	FPKM
**122**	445.5	18.25	Trimmomatic	RUM	Stringtie	Coverage
**123**	448	4.25	BBDuk	STAR	eXpress	TPM
**124**	449.5	4.5	BBDuk	Bowtie2	eXpress	FPKM
**125**	452	12.75	Trimmomatic	HiSat2	HTSeq INTER	Raw
**126**	455.5	13.5	Trimmomatic	HiSat2	HTSeq UNION	Raw
**127**	456	15.25	BBDuk	RUM	Stringtie	Coverage
**128**	459	4	Trimmomatic	Bowtie2	eXpress	TPM
**129**	462	3.5	BBDuk	Bowtie2	eXpress	TPM

**130**	462	10.75	Cutadapt	Bowtie2	eXpress	FPKM
**131**	467	15.75	Trimmomatic	RUM	HTSeq INTER	Raw
**132**	471.5	14.25	Trimmomatic	HiSat2	Stringtie	Coverage
**133**	472	16.5	BBDuk	Bowtie2	eXpress	Eff_Counts
**134**	472.5	15	Trimmomatic	RUM	HTSeq UNION	Raw
**135**	474	17.5	Trimmomatic	TopHat2	Stringtie	Coverage
**136**	481	14.25	Trimmomatic	STAR	eXpress	Eff_Counts
**137**	482	12	BBDuk	RUM	HTSeq INTER	Raw
**138**	485	12.5	BBDuk	Bowtie2	eXpress	Est_Count
**139**	486.5	22.5	Trimmomatic	Bowtie2	eXpress	Eff_Counts
**140**	488	2.5	BBDuk	TopHat2	Stringtie	Coverage
**141**	488.5	3	BBDuk	HiSat2	Stringtie	Coverage
**142**	489	11.25	Trimmomatic	Salmon-FMD-ISR		NumReads
**143**	489	13.25	Trimmomatic	STAR	eXpress	Est_Count
**144**	491.5	11	Trimmomatic	Salmon-FMD-AUT		NumReads
**145**	492.5	12	BBDuk	RUM	HTSeq UNION	Raw
**146**	493	16.5	Trimmomatic	Kallisto		Est-Counts
**147**	494.5	6	Cutadapt	Bowtie2	eXpress	TPM
**148**	495.5	6	BBDuk	STAR	eXpress	Eff_Counts
**149**	496.5	6.5	BBDuk	HiSat2	HTSeq INTER	Raw
**150**	497.5	2.5	BBDuk	STAR	Stringtie	Coverage
**151**	497.5	11.25	Trimmomatic	Salmon-QMB-ISR		NumReads
**152**	501	14	Trimmomatic	STAR	HTSeq INTER	Raw
**153**	501.5	16.5	Trimmomatic	STAR	Stringtie	Coverage
**154**	502	12.25	Trimmomatic	TopHat2	HTSeq INTER	Raw
**155**	503.5	7.75	BBDuk	HiSat2	HTSeq UNION	Raw
**156**	504	12.5	Trimmomatic	TopHat2	HTSeq UNION	Raw
**157**	504	13.75	Trimmomatic	STAR	HTSeq UNION	Raw
**158**	504.5	4	BBDuk	STAR	HTSeq INTER	Raw
**159**	510.5	5.5	BBDuk	STAR	HTSeq UNION	Raw
**160**	511	5.5	BBDuk	STAR	eXpress	Est_Count
**161**	513	7.75	BBDuk	TopHat2	HTSeq INTER	Raw
**162**	513	9	BBDuk	Salmon-QMB-AUT		NumReads
**163**	513.5	8.25	BBDuk	Sailfish-ISR		NumReads
**164**	514	8.25	BBDuk	Salmon-QMB-ISR		NumReads
**165**	514.5	11.75	BBDuk	Salmon-FMD-ISR		NumReads
**166**	515	8.5	BBDuk	TopHat2	HTSeq UNION	Raw
**167**	515	12.5	BBDuk	Salmon-FMD-AUT		NumReads
**168**	515.5	8	BBDuk	Kallisto		Est-Counts
**169**	516.5	23	Trimmomatic	Bowtie2	eXpress	Est_Count
**170**	534	4.5	Cutadapt	STAR	eXpress	Est_Count
**171**	538	6.5	Cutadapt	HiSat2	HTSeq INTER	Raw
**172**	539.5	7	Cutadapt	HiSat2	HTSeq UNION	Raw
**173**	540.5	5	Cutadapt	RUM	Stringtie	Coverage
**174**	552.5	2.5	Cutadapt	HiSat2	Stringtie	Coverage

**175**	553.5	1.25	Cutadapt	Bowtie2	eXpress	Est_Count
**176**	554	11.75	Cutadapt	Salmon-QMB-ISR		NumReads
**177**	555	11.25	Cutadapt	Sailfish-ISR		NumReads
**178**	555.5	11.75	Cutadapt	Salmon-QMB-AUT		NumReads
**179**	556	7	Cutadapt	STAR	eXpress	Eff_Counts
**180**	556.5	9.75	Cutadapt	Kallisto		Est-Counts
**181**	556.5	14	Cutadapt	Salmon-FMD-ISR		NumReads
**182**	557	14.5	Cutadapt	Salmon-FMD-AUT		NumReads
**183**	560.5	0.5	Cutadapt	RUM	HTSeq INTER	Raw
**184**	560.5	1	Cutadapt	RUM	HTSeq UNION	Raw
**185**	562	3	Cutadapt	TopHat2	Stringtie	Coverage
**186**	566.5	3.75	Cutadapt	STAR	Stringtie	Coverage
**187**	568.5	3.5	Cutadapt	Bowtie2	eXpress	Eff_Counts
**188**	572	5.25	Cutadapt	TopHat2	HTSeq INTER	Raw
**189**	573	2.5	Cutadapt	STAR	HTSeq UNION	Raw
**190**	573.5	2.5	Cutadapt	STAR	HTSeq INTER	Raw
**191**	578	4.5	Cutadapt	TopHat2	HTSeq UNION	Raw
**192**	593.5	13.5	Trimmomatic	Salmon-QMB-AUT		NumReads

Supplementary Table S7. Results of the trimming algorithms. Percentage of surviving reads and alignment rate for each of the analysed samples.

Sample*	Percentage of surviving reads			Alignment rate (%)**		
	Trimmomatic	Cutadapt	BBDuk	Trimmomatic	Cutadapt	BBDuk
CLA-T0-S1	83.5	95.9	92.7	94,9 ± 0,4	90,5 ± 3,1	97,0 ± 1,2
CLA-T0-S 2	84.4	95.0	88.8	96,9 ± 0,8	95,0 ± 1,9	98,0 ± 0,7
CLA-T0-S3	84.2	95.4	89.6	96,8 ± 1,6	94,0 ± 2,1	97,9 ± 1,1
CLB-T0-S1	88.9	96.8	93.9	96,1 ± 0,5	94,0 ± 2,4	97,6 ± 0,8
CLB-T0-S2	79.4	94.5	85.6	95,8 ± 1,2	93,8 ± 2,5	97,3 ± 1,4
CLB-T0-S3	83.2	95.6	89.9	96,0 ± 1,1	93,1 ± 2,6	97,8 ± 0,8
**Median CLA**	**84.2**	**95.4**	**89.6**	**96,1 ± 1,4**	**93,1 ± 2,6**	**97,4 ± 1,3**
**Median CLB**	**83.2**	**95.6**	**89.9**	**96,0 ± 1,0**	**93,8 ± 2,5**	**97,6 ± 1,0**
**Overall median**	**83.8**	**95.5**	**89.8**	**96,1 ± 1,2**	**93,4 ± 2,5**	**97,5 ± 1,2**

*CLA = Cell line A (KMS12-BM); CLB = Cell line B (JJN-3); T0 = DMSO

** Values are represented as median ± median absolute deviation (MAD)

Suppplementary Table S8. Kruskal-Wallis and Dunn’s test results for raw gene expression quantification based on the trimming algorithms.

Contrast	Chi squared statistic	Degrees of freedom	*p*-value	Pairwise contrast	Dunn's pairwise z test statistic	Dunn's test FDR
Kruskal-Wallis	3.07E+00	2	2.20E-01	Trimmomatic vs. Cutadapt	1.59E+00	1.69E-01
				Trimmomatic vs. BBDuk	1.50E-01	4.40E-01
				Cutadapt vs. BBDuk	-1.44E+00	1.13E-01

Suppplementary Table S9. Kruskal-Wallis and Dunn's test results for raw gene expression quantification based on the alignment algorithms.

Contrast	Chi squared statistic	Degrees of freedom	*p*-value	Pairwise contrast	Dunn's pairwise z test statistic	Dunn's test FDR
Kruskal-Wallis	3.85E+00	4	4.30E-01	Bowtie2 vs. HiSat2	1.63E+00	2.60E-01
				Bowtie2 vs. RUM	1.87E+00	3.10E-01
				HiSat2 vs. RUM	2.77E-01	4.89E-01
				Bowtie2 vs. STAR	1.52E+00	2.14E-01
				HiSat2 vs. STAR	-2.76E-01	4.35E-01
				RUM vs. STAR	-5.83E-01	4.66E-01
				Bowtie2 vs. Tophat2	1.31E+00	2.38E-01
				HiSat2 vs. Tophat2	-3.66E-01	5.10E-01
				RUM vs. Tophat2	-6.43E-01	5.20E-01
				STAR vs. Tophat2	-1.30E-01	4.48E-01

Suppplementary Table S10. Kruskal-Wallis and Dunn's test results for raw gene expression quantification based on the counting method.

Contrast	Chi squared statistic	Degrees of freedom	*p*-value*	Pairwise contrast	Dunn's pairwise z test statistic	Dunn's test FDR*
Kruskal-Wallis	3.38E+01	5	< 0.01	Cufflinks vs. eXpress	-4.22E+00	6.15E-05
				Cufflinks vs. HTSeq INTER	-1.08E+00	2.11E-01
				eXpress vs. HTSeq INTER	4.30E+00	6.47E-05
				Cufflinks vs. HTSeq UNION	-1.02E+00	2.09E-01
				eXpress vs. HTSeq UNION	4.36E+00	9.56E-05
				HTSeq INTER vs. HTSeq UNION	7.43E-02	4.70E-01
				Cufflinks vs. RSEM	-2.91E-01	4.13E-01
				eXpress vs. RSEM	3.88E+00	1.94E-04
				HTSeq INTER vs. RSEM	7.19E-01	2.95E-01
				HTSeq UNION vs. RSEM	6.67E-01	2.91E-01
				Cufflinks vs. Stringtie	-2.69E+00	1.08E-02
				eXpress vs. Stringtie	2.26E+00	1.99E-02
				HTSeq INTER vs. Stringtie	-2.28E+00	2.13E-02
				HTSeq UNION vs. Stringtie	-2.35E+00	2.33E-02
				RSEM vs. Stringtie	-2.33E+00	2.12E-02

* Statistically significant comparisons (< 0.05) are shown in green

Suppplementary Table S11. Kruskal-Wallis and Dunn’s test results for raw gene expression quantification based on the normalization approaches.

Contrast	Chi squared statistic	Degrees of freedom	*p*-value*	Pairwise contrast	Dunn's pairwise z test statistic	Dunn's test FDR*
Kruskal-Wallis	1.51E+02	8	< 0.01	Coverage vs. Eff_Counts	-7.65E-02	4.97E-01
				Coverage vs. Est_Count	-3.86E-01	4.50E-01
				Eff_Counts vs. Est_Count	-2.50E-01	4.81E-01
				Coverage vs. FPKM	3.87E+00	1.09E-04
				Eff_Counts vs. FPKM	3.01E+00	2.23E-03
				Est_Count vs. FPKM	3.92E+00	1.01E-04
				Coverage vs. NumReads	-4.94E-01	4.30E-01
				Eff_Counts vs. NumReads	-3.17E-01	4.66E-01
				Est_Count vs. NumReads	-4.98E-02	4.80E-01
				FPKM vs. NumReads	-4.82E+00	2.36E-06
				Coverage vs. Raw	-4.18E-01	4.51E-01
				Eff_Counts vs. Raw	-2.40E-01	4.71E-01
				Est_Count vs. Raw	5.76E-02	4.91E-01
				FPKM vs. Raw	-5.45E+00	9.82E-08
				NumReads vs. Raw	1.32E-01	4.88E-01
				Coverage vs. RLE	4.74E+00	3.27E-06
				Eff_Counts vs. RLE	3.75E+00	1.58E-04
				Est_Count vs. RLE	4.72E+00	3.28E-06
				FPKM vs. RLE	1.46E+00	1.04E-01
				NumReads vs. RLE	5.67E+00	4.33E-08
				Raw vs. RLE	6.31E+00	9.93E-10
				Coverage vs. TMM	6.94E+00	2.36E-11
				Eff_Counts vs. TMM	5.46E+00	1.08E-07
				Est_Count vs. TMM	6.71E+00	8.62E-11
				FPKM vs. TMM	4.42E+00	1.21E-05
				NumReads vs. TMM	8.03E+00	8.43E-15
				Raw vs. TMM	9.01E+00	3.69E-18
				RLE vs. TMM	2.70E+00	5.45E-03
				Coverage vs. TPM	3.84E+00	1.14E-04
				Eff_Counts vs. TPM	2.97E+00	2.43E-03
				Est_Count vs. TPM	3.89E+00	1.06E-04
				FPKM vs. TPM	-1.37E-01	5.01E-01
				NumReads vs. TPM	4.82E+00	2.59E-06
				Raw vs. TPM	5.50E+00	1.00E-07
				RLE vs. TPM	-1.63E+00	7.81E-02
				TMM vs. TPM	-4.67E+00	3.88E-06

* Statistically significant comparisons (p-value or FDR < 0.05) are shown in green

Suppplementary Table S12. Kruskal-Wallis and Dunn's test results for raw gene expression quantification based on the pseudoalignment algorithms.

Contrast	Chi squared statistic	Degrees of freedom	*p*-value	Pairwise contrast	Dunn's pairwise z test statistic	Dunn's test FDR
Kruskal-Wallis	1.08E+00	2	5.80E-01	Kallisto *vs.* Sailfish	8.08E-01	3.14E-01
				Kallisto *vs.* Salmon	1.02E+00	4.63E-01
				Sailfish *vs.* Salmon	-4.33E-03	4.98E-01

Suppplementary Table S13. Kruskal-Wallis and Dunn’s results for the pipelines based on pseudoaligner and classic aligner data.

Contrast	Chi squared statistic	Degrees of freedom	*p*-value	Pairwise contrast	Dunn's pairwise z test statistic	Dunn's test FDR
Kruskal-Wallis	9.51E+00	7	2.20E-01	Bowtie2 vs. HiSat2	1.58E+00	2.00E-01
				Bowtie2 vs. Kallisto	-6.64E-01	3.94E-01
				HiSat2 vs. Kallisto	-1.75E+00	2.79E-01
				Bowtie2 vs. RUM	1.76E+00	3.63E-01
				HiSat2 vs. RUM	2.14E-01	4.65E-01
				Kallisto vs. RUM	1.88E+00	4.25E-01
				Bowtie2 vs. Sailfish	2.65E-02	5.08E-01
				HiSat2 vs. Sailfish	-1.02E+00	3.06E-01
				Kallisto vs. Sailfish	5.64E-01	4.01E-01
				RUM vs. Sailfish	-1.15E+00	2.70E-01
				Bowtie2 vs. Salmon	2.93E-02	5.26E-01
				HiSat2 vs. Salmon	-1.68E+00	2.58E-01
				Kallisto vs. Salmon	7.06E-01	3.96E-01
				RUM vs. Salmon	-1.89E+00	8.30E-01
				Sailfish vs. Salmon	-7.39E-03	4.97E-01
				Bowtie2 vs. STAR	1.50E+00	2.09E-01
				HiSat2 vs. STAR	-2.44E-01	4.71E-01
				Kallisto vs. STAR	1.68E+00	2.18E-01
				RUM vs. STAR	-4.81E-01	4.20E-01
				Sailfish vs. STAR	9.26E-01	3.31E-01
				Salmon vs. STAR	1.62E+00	2.11E-01
				Bowtie2 vs. Tophat2	1.19E+00	2.71E-01
				HiSat2 vs. Tophat2	-4.44E-01	4.18E-01
				Kallisto vs. Tophat2	1.50E+00	1.89E-01
				RUM vs. Tophat2	-6.57E-01	3.76E-01
				Sailfish vs. Tophat2	7.68E-01	3.87E-01
				Salmon vs. Tophat2	1.27E+00	2.61E-01
				STAR vs. Tophat2	-2.48E-01	4.89E-01
